# Obtaining accurate population estimates with reduced workload and lower fish mortality in multi-mesh gillnet sampling of a large pre-alpine lake

**DOI:** 10.1371/journal.pone.0299774

**Published:** 2024-03-18

**Authors:** Steffen Bader, Julia Gaye-Siessegger, Barbara Scholz, Mário Mota-Ferreira, Alexander Brinker

**Affiliations:** 1 Fisheries Research Station Baden-Württemberg, Langenargen, Germany; 2 The Bavarian State Institute of Forestry, Freising, Germany; 3 University of Konstanz, Konstanz, Germany; Central University of South Bihar, INDIA

## Abstract

The EU Water Framework Directive requires monitoring of the ecological status of lakes, with fish as a relevant class of biotic quality indicator, but monitoring fish populations in large lakes is demanding. This study evaluated use in Lake Constance of a novel multi-mesh gillnet modified to reduce catch numbers. In direct comparison with conventional European Committee for Standardization (CEN) nets we achieved 48% reduction in fish mortality with 38% less labour for tasks directly influenced by fish catch numbers, while maintaining comparable species composition and catch per unit effort. Comparison of mesh sizes indicated no significant reduction in species detection in area-reduced panels of the small mesh sizes, while total observed species richness was greater when using the modified nets. Differences in benthic species communities among depth strata were common, while those of pelagic zones were more homogeneous and did not differ significantly with depth. Catches of different net types from the same depth stratum did not exhibit significant differences. The dominance structure of the most common species, relevant to lake assessment, was similar in catches of both net types, suggesting overall superiority of the modified nets in Lake Constance. Sampling conducted according to standard European CEN protocol, while deploying 60% fewer nets, yielded sufficiently precise abundance estimates for monitoring shallow areas of the benthic zone. A 50% difference in the abundance of dominant species was detected among sampling events with a certainty of 95%. The sample did not provide comparable accuracy in deep benthic strata or the pelagic zone, but was adequate to record complete inventories of species present. Based on this trial data, a new stratified sampling design is proposed for monitoring large lake fish communities for ecological assessment. Depth-dependent fish communities were used to calculate the required number of nets, which resulted in a 69% reduction for the entire lake compared to the CEN calculation method. Using the modified nets increases the feasibility of performing WFD surveys, by reducing effort and cost, while the simultaneous halving of fish mortality minimises the negative impact of fish surveys.

## Introduction

The increasing human population places ever greater demands on ecosystems [1], especially on freshwater bodies, the most threatened of aquatic habitats [2] and one that is subject to the requirements of many stakeholders. In addition to the impact of climate change [3–5], widespread anthropogenic pressures on lakes include eutrophication, habitat degradation, introduction of alien species, and pollution [6]. The protection and restoration of such systems requires management based on robust documentation of the *status quo* and, within the European Union, compliance with the preconditions for the preservation and enhancement of the ecological status of surface waters set out in the Water Framework Directive (WFD) [7]. The WFD obliges member states to achieve good ecological status of surface waters by 2027. For lakes ≥50 ha, assessment of hydromorphological and chemical conditions is based largely on four biotic components: pelagic phytoplankton, macrophytes, macroinvertebrates, and fish. The longevity and mobility of fish and their range of trophic levels make them robust temporal and spatial indicators of entire ecosystem health and function [8–10] that respond predictably to disruptions such as eutrophication [11–13], habitat destruction and fragmentation [14], acidification [15], and climate change [3].

While representative samples of fish populations in small streams and ponds can be collected with comparative ease, obtaining accurate and precise data regarding fish stock of large lakes and reservoirs can be challenging [16–19]. The basis of fish stock assessment is sampling, with accuracy and precision depending chiefly on the efficacy of the equipment and the nature of the sampling campaign [17]. For the assessment of fish communities in lakes, a variety of gear and sampling strategies have been devised (e.g., gillnets, trawls, electro-fishing, hydroacoustics) and applied for specific conditions and objectives [20–22], each with advantages and disadvantages. In entire lake fish stock assessment, it is usually appropriate to use a combination of sampling techniques [17, 19]. In many EU member states, monitoring is conducted using a combination of electric and gillnet fishing [23], with the most common gillnet method based on a Scandinavian system [24, 25] developed by Appelberg [20] and later standardized as DIN 14757 [26]. This multi-mesh gillnet sampling design can fulfil the WFD (Annex V) requirement for estimates of lake species diversity, relative abundance, and biomass expressed as catch-per-unit-effort (CPUE) and body length or age structure [19]. However, gillnets are passive sampling gear, and do not ensure coverage of a given area/water volume or provide quantitative estimates of abundance or biomass [27, 28]. The European standard CEN protocol [26] is conducted using horizontal nets deployed separately in the benthic and pelagic zones. The effort required within each zone depends on lake size and depth and consists of random sampling with multiple replicates at a given depth strata.

Compared to techniques such as trammel nets, trawls, seines, or fyke nets, use of multi-mesh gillnets is considered relatively robust. Bagenal [29] found that gillnets produced the lowest intra-sample variance, and Jensen [30] reported an accurate estimate of tagged fish in a perch population. However, catch efficacy depends to a great extent on fish behaviour and therefore on species-specific attributes and environmental conditions. Comparatively consistent factors such as morphology, trophic status, and community composition can influence the utility of the applied method and equipment type within a given lake [18]. In addition, fish distribution within a lake is not random, but dependent on species and ontogeny. It varies in space and time, being subject to inconsistent physical (e.g., temperature, light), chemical, and biotic factors (e.g., phytoplankton density, predator activity) linked to season and time of day. Sampling methods should, therefore, be standardized to minimize bias whenever possible as well as verified for use in lakes with conditions differing from those in which the technique is developed. The general applicability and appropriateness of a sampling campaign should be scrutinized before analyses are carried out and conclusions drawn.

How far the sample-based depiction of a fish community deviates from the real-life situation can be difficult to assess. Multiple sampling methods yielding similar results can increase confidence [17], but when techniques are not directly comparable, variation in the estimated abundance of common species might indicate heterogeneity of fish assemblage in a lake or point to poor design of the sampling campaign. In the case of DIN 14757, the precision of a single sampling event must be sufficient to ensure detection of a 50% difference from other samplings in the abundance of dominant species with 95% likelihood. Despite this requirement, few studies report the precision of sampling campaigns [18, 31], an exception being those dealing with the standardization of net sampling by comparing abundance of captured species among sites, populations, or sampling events [18, 32, 33]. Degermann [31] and Holmgren [34] calculated the precision of a single sampling event by assessing variation among single net catches within entire lakes or within a depth stratum.

Since species richness is related to lake area [35], and fish density is not homogenous within a lake, the required sampling effort is related to lake size [31]. For small lakes, the number of nets required under the CEN protocol is manageable, and multi-mesh gillnet sampling can be relatively time- and cost-effective [18, 36]. However, the DIN 14757 reaches its limits in large deep-water bodies like Lake Constance, where the number of nets and the effort required to deploy them render the method logistically challenging and prohibitively expensive [37]. Gillnet sampling is also highly invasive, with the majority of captured individuals not surviving [38], making it ethically and environmentally unacceptable.

To reduce the per net ecological impact and the labour associated with scientific monitoring, we compared the performance of a novel modified gillnet design (MOD) with the standard CEN net. Modifications were based on general net sampling experiences in Central European lakes where high catch of small fish are common and fish numbers naturally decline with size [39]. For example, Alexander et al. [40] showed that, in 17 pre-alpine, subalpine, and alpine European lakes, the majority of fish caught with CEN nets were smaller than 10 cm. Lake Constance is representative of these lake types. The MOD nets were designed to reduce large but redundant catches of fish of small size classes by decreasing the area of small mesh sizes. As fish of larger size classes are rare in these lake types, they might be underrepresented as an effect of the small areas of CEN nets. Therefore, the panels of the large mesh sizes were expanded in the MOD nets.

The study compared fish mortality with the use of MOD nets with that of conventional CEN nets in Lake Constance. Since the record of species depends directly on the fishing effort [41] and thus on net area, a reduction in species record with the use of MOD nets is a possibility. In addition, change in the net conformation can result in an altered guiding effect and thus affect the catch composition.

Despite the potential obstacles, the hypothesis explored in this study is that the catch species composition and numbers per unit effort (NPUE, individuals per 100 m^2^ net area) using MOD nets will not show relevant differences from monitoring with CEN nets.

The assessment of the ecological status of water bodies involves detecting, not only composition, abundance, and age structure of the fish population, but also, for example, the corresponding guilds (spawning, habitat). Especially in lakes such as Lake Constance, assessment data may be impacted by the lack of detection of rare species or related guilds not regularly found in surveys. Such discrepancies also occur as a result of annual variations and can only be counteracted by long-term monitoring. For this reason, when assessing lake fish communities, netting data is usually supplemented with information from other sources [42]. When evaluating performance of the MOD nets, the total number of recorded species must be comparable to that found with CEN nets and the proportions of common species should show similar distribution.

We also calculated the reduction in labour related to the lower catch in the MOD nets in Lake Constance.

In a second part of this study, we evaluated the sampling campaign, testing the accuracy of species richness and abundance estimates obtained with the modified nets compared to CEN and the smaller sample size than recommended by the CEN protocol. Since the sampling is to be carried out for the WFD, the methods specified in the DIN 14757 were used to calculate precision.

A third part of the study used the results gained from the sampling campaign to inform the design of a new stratified sampling protocol for fish communities in Lake Constance and similar lakes. Since the monitoring serves to evaluate the lake for the WFD, the number of nets required to reliably estimate abundance of dominant species were calculated according to Pringle [43], which is the basis for calculating required net numbers in the CEN protocol [26]. We compared the required net numbers calculated according to Pringle [43] to an alternative approach based on a power t-test to detect differences in catches.

## Methods

Approval of the present study by a review board institution or ethics committee was not necessary, all fish were caught under permits issued by the authorized fisheries administrations. All fish were caught according to the German Animal Protection Law (Tierschutzgesetz § 4) and the ordinance on slaughter and killing of animals (Tierschutzschlachtverordnung § 13).

### Sampling area

Lake Constance is a large deep lake with shoreline in Austria, Germany, and Switzerland (Table 1), classified by Riedmüller et al. [45] as calcareous, stratified, and alpine, but widely described in scientific literature as pre-alpine [46]. Its largest tributary is the River Rhine, which drains from the Alps and is characterized by cold water and, occasionally, an abundance of suspended sediment. The river runs through the lake, and a three-kilometre section connects Upper Lake Constance (ULC) with the smaller and shallower Lower Lake Constance (LLC). The status of Lake Constance is oligotrophic to mesotrophic, with total phosphorus concentrations (volume-weighted annual mean) of 6.2 and 13.1 μg *L*^-1^ in ULC and LLC, respectively [47]. The lake is monomictic, and prior to 1990 was home to at least 30 fish species, a comparatively high count [48].

**Table 1:** Physical features of Upper Lake Constance and Lower Lake Constance [44].

	Upper Lake Constance	Lower Lake Constance
Surface area [km^2^]	472	62
Volume [km^3^]	47.6	0.8
Depth max. [m]	253	47
Depth mean [m]	101	13
Secci depth (pelagic zone) [m]	5.2*	5.8**
Catchment area [km^2^]	10,919	555
Water retention time [yr]	4.3	0.08
Average surface water temp. 2019 [°C]	13	14

*September 2019

**October 2019 [47]

Fishing with nets has taken place in Lake Constance since at least the Neolithic [49], and has been an important economic activity that has demanded regulation from authorities since the 14th century [50]. Nonetheless, the number of licensed fishermen has been decreasing since 1931, and tourism is now the main socio-economic activity related to Lake Constance [51]. At least 4 million people rely on Lake Constance as a source of drinking water [52]. Lake Constance underwent pronounced eutrophication with the last century but international efforts have been able to revert to its past trophic state [51]. As result of increasing nutrient levels, fisheries yields varied between a 10-yearaverage of 423 metric tons (mt) before 1955 to over than 1000 mt between 1956 and 1990. Though fishers benefitted from increased productivity during an initial mesotrophic phase, these advantages were effectively neutralized when eutrophication became severe, as yields of the most profitable species decreased, and the eutrophication-induced hypoxia led to the probable extinction of an endemic dwarf whitefish species [48, 51]. Since reoligotrophication fishery yields have continued to diminish and are, strikingly, now even below pre-1950 levels [51]. As a result of the sharply declining catch of whitefish, a three year fishing ban on whitefish in Lake Constance will be imposed from January 2024 [53].

### Gillnetting

**Fig 1:**
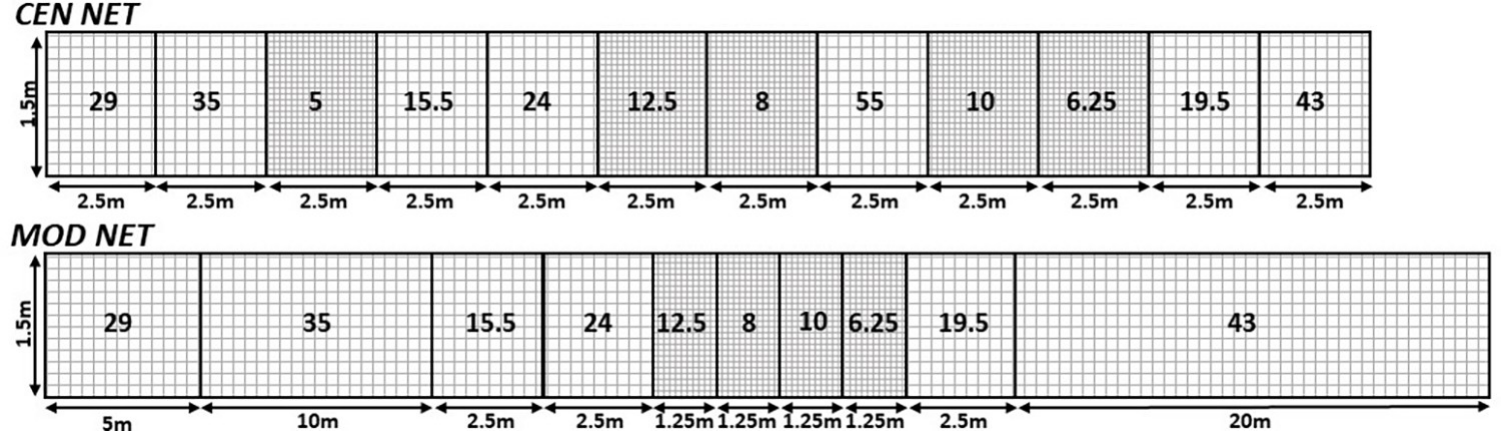
Standard CEN and modified (MOD) benthic gill nets including length of mesh size segments.

Sampling was conducted using either standard multi-mesh gillnets [26] or the novel MOD nets. The MOD nets lacked the smallest and largest mesh sizes (5 mm and 55 mm) of CEN nets (Fig 1). The 5 mm mesh size was eliminated to reduce large but redundant catches of fish of small size classes and the 55 mm mesh size as the few fish of the size and age classes usually caught in this mesh size rarely provide valuable information in lake assessment. The mesh-size areas of the MOD nets differed from those of CEN nets in which the length of each mesh-size panel is 2.5 m (3.75 m^2^ for benthic nets and 15 m^2^ for pelagic nets). In the MOD nets, panel sizes of small mesh (6.25, 8, 10, and 12.5 mm) were half those of the standard CEN net, while the panel sizes of large mesh were two- (29 mm), four- (35 mm), and eight-fold (43 mm) the CEN area. The sequence of mesh-size panels was similar in the net types, and the segments in benthic and pelagic MOD nets were of identical length. Net height in benthic and pelagic settings was 1.5 m and 6 m, respectively, for both CEN and MOD types.

**Fig 2:**
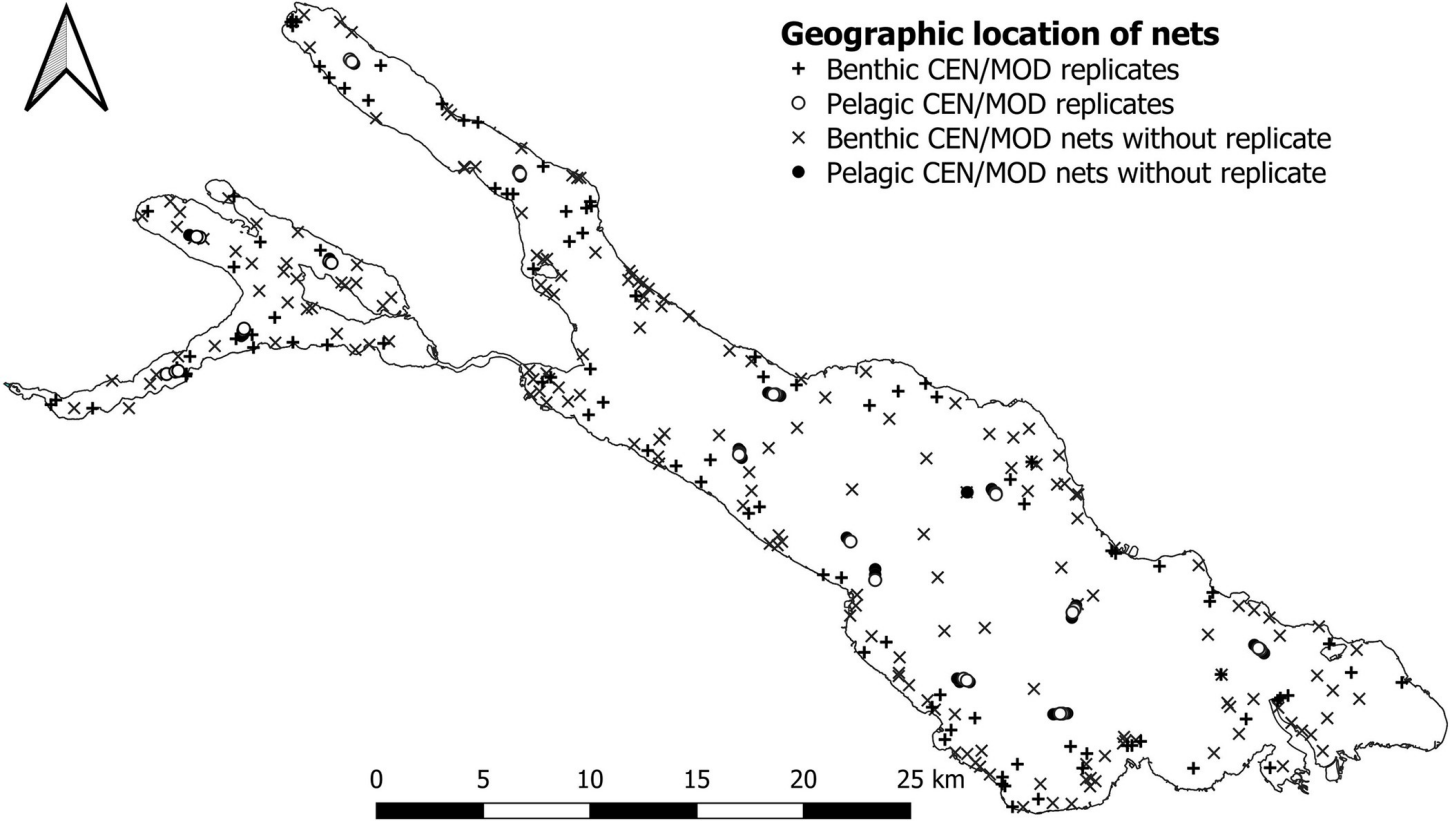
Sampling sites in ULC and LLC. Benthic and pelagic sites with and without CEN/MOD replicates are indicated. The map of Lake Constance derived from „Umweltinformationssystem (UIS) der LUBW Landesanstalt für Umwelt Baden-Württemberg" (https://udo.lubw.baden-wuerttemberg.de/public/index.xhtml;jsessionid=C1E1293728E2675A6AF2CF5BD5E0255B) under user agreement https://www.lubw.baden-wuerttemberg.de/umweltinformationssystem/nutzungsvereinbarung (in German), last accessed November 29, 2023. Sampling sites were individually redrawn using GIS software.

To compare performance, CEN and MOD nets were deployed in pairs. One CEN and one MOD net were set at the same orientation to the shore at a sampling site on the same night. We deployed 81 benthic and 26 pelagic CEN/MOD replicates in ULC and 20 benthic and nine pelagic in LLC (Fig 2, Table 2).

**Table 2:** Number of nets within depth strata of ULC and LLC. Setting depth was always >1.5 m. Net numbers of CEN/MOD replicates used for net type comparisons are shown in brackets.

	Upper Lake Constance				Lower Lake Constance			
Depth stratum [m]	Benthic zone		Pelagic zone		Benthic zone		Pelagic zone	
	CEN	MOD	CEN	MOD	CEN	MOD	CEN	MOD
0–2.9	9 (9)	30 (9)	-	-	3 (3)	10 (3)	-	-
3–5.9	11 (11)	30 (11)	-	-	4 (4)	10 (4)	-	-
0–5.9	-	-	-	12	-	-	-	4
6–11.9	12 (12)	30 (12)	2 (2)	12 (2)*	4 (4)	10 (4)	3 (3)	4 (3)
12–19.9	11 (11)	30 (11)	12 (12)	12 (12)	3 (3)	10 (3)	4 (4)	4 (4)
20–34.9	10 (10)	30 (10)	12 (12)	12 (12)	3 (3)	10 (3)	2 (2)	2 (2)*
35–49.9	8 (8)	30 (8)	-	12	3 (3)	6 (3)	-	-
50–74.9	9 (9)	12 (9)	-	12	-	-	-	-
75–99.9	11 (11)	13 (11)	-	-	-	-	-	-
100–149.9	-	9	-	-	-	-	-	-
150–199.9	1	8	-	-	-	-	-	-
200–250	1	5	-	-	-	-	-	-
Sum	83 (81)	227 (81)	26 (26)	72 (26)	20 (20)	56 (20)	9 (9)	14 (9)
**Total**	**408**				**99**			

*Excluded from PERMDISP test

Sampling was conducted over four-night periods from September 12, 2019 through October 07, 2019. Each site was fished only once. Nets were set from three boats, each operated by two fishery personnel. Except for the reduced number of nets deployed in the benthic zone, nets were set according to the CEN standard protocol [26]. Each lake basin was divided into depth strata and sampling was conducted randomly within each stratum. If possible, all sampling nights recovered samples from all strata.

According to CEN protocol, routine monitoring of Lake Constance would require setting 605 benthic nets in ULC and an additional 80 in LLC, incurring prohibitively high costs and unacceptable fish mortality. In this study, the benthic sampling involved 83 CEN nets and 227 MOD nets in ULC and 20 CEN nets and 56 MOD in LLC. Pelagic nets were set according to CEN protocol at 12 sites in ULC including 26 CEN and 72 MOD nets covering depth strata from 0–75 m. In LLC, nine CEN nets and 14 MOD nets were set at four sites and covered depth strata from 0–35 m (Fig 2, Table 2). Nets were set before sunset and lifted after dawn (~14 hours) in order to cover both nycthemeral migration periods [54].

The examination of lifted nets and disentanglement of the captured fish was carried out by two teams of three trained personnel. Another team of two experts identified fish to species, with the exception of the three difficult to distinguish coregonids inhabiting ULC [*Coregonus wartmanni* (Bloch 1784), *Coregonus arenicolus* Kottelat 1997, *Coregonus macrophthalmus* Nüsslin 1882], which were grouped as ‘whitefish’. Fish were measured to 1 mm total length and weighed to the nearest 10 g (wet weight).

### Statistical analysis

As two types of net were used, NPUE was calculated as fish per 100 m^2^ net area for each mesh size. The NPUE for an entire net was defined as the sum of NPUE for all mesh size panels. In order to avoid potential inaccuracies resulting from division by the unequal number of mesh panels in the two net types, comparisons relating to catch efficacy were limited to mesh sizes present in both. Since the objective of this study was to evaluate sampling efficacy rather than the representation of actual species proportions within whole lake assemblages, NPUE was not weighted for lake volume. As net types were set in pairs, there is an argument for using matched-pair statistics when comparing them, however, because of the extremely patchy distribution of most fish species, sometimes resulting in extremes of zero catch in one net and mass catch in the other, we opted for unpaired tests. The two lake basins were treated separately in the analyses.

### Comparison of CEN and MOD nets

The comparison of CEN and MOD nets included only catch data from sampling sites with replicates of CEN and MOD nets.

*Species richness and NPUE*. Equivalence tests using confidence intervals were conducted to assess whether CEN and MOD nets showed similar numbers of species and NPUE. An upper (ΔU) and lower (−ΔL) equivalence limit was specified based on the smallest effect-size of interest. Equivalence was accepted if the 90% confidence interval of differences fell within the predefined equivalence range: -ΔL < 90 CI < ΔU. The range was defined as +/- 50% of the mean difference. Although this was sufficient to claim equivalence, we show additional results for a range of +/- 20% of the mean. The power achieved by the number of samples was calculated for each equivalence range. The number of samples required to reach a power of 0.8 is also given. Calculations were performed using the TOSTER package [55] in R software [56]. Comparisons were made for each mesh size separately, including nets from depth strata 0–100 m. Benthic and pelagic samples were analysed separately.

A generalized linear mixed model (GLMM) was used to test whether the use of MOD nets led to number of species and NPUE significantly different from CEN nets. Fixed factors were α = net type, β = depth strata, γ = zone, δ = mesh size and an interaction between net type and depth strata (α * β); and, as random factors: ε = day and ζ = net. Prior to analysis, the NPUE data was log(x+0.1) transformed. In the case of number of species, the residuals were not normally distributed. Poisson distribution and log as the link function was used. Analyses were performed with JMP Pro 16. The GLMM was run using the add-in provided by Dong (JMP Genomics Intern).

*Catch composition*. We used non-metric multidimensional scaling (nMDS, PRIMER® 7, PRIMER-E Ltd, Ivybridge) for comparing catch composition (species-specific NPUE) between zones (benthic/pelagic), depth strata, and net types [57]. The ordination method was based on a Bray-Curtis similarity matrix that included a dummy (value = 1) using log(x+1)-transformed NPUE values of each net. We used a permutation analysis of multidimensional dispersion with 999 permutations and distances to the centroid (PERMDISP, PRIMER ® 7, PRIMER-E Ltd, Ivybridge) to test whether fish community structure revealed by CEN net sampling varied significantly from that found with MOD nets [58]. Sample groups were generated according to zone, depth stratum, and net type. Groups with fewer than three CEN/MOD pairs were excluded from the analysis. Mean distances to group centroids (±1 standard deviation) were plotted for CEN and MOD net comparisons [59].

We conducted further PERMDISP analysis with 999 permutations to test whether the composition [species and number of individuals as mean *z*-values = distance-to-centroid of each observation in the Bray-Curtis matrix of log(x+1) transformed NPUE data] of benthic and pelagic catches differed and should be evaluated separately in subsequent analyses. We also tested for differences in the catch composition of CEN and MOD net types within and among depth strata using one-way analysis of similarities with 999 permutations (ANOSIM, PRIMER ® 7, PRIMER-E Ltd, Ivybridge) [60].

*Labour*. Labour was calculated based on the mesh size-dependent catch of CEN and MOD nets. The labour required for tasks such as disentanglement of captured fish and catch documentation is directly dependent on catch quantity. Therefore, the analysed workload was limited to these steps. The time required for further work such as setting and lifting of the nets is waterbody-specific and not influenced by net type.

### Effectiveness of the sampling campaign

*Species richness*. To examine the relationship of fishing effort to estimated species richness, species accumulation curves [61, 62] were obtained using the SPECCACUMfunction [63] of the ‘vegan’ package in R [55]. This provided a good estimate of biodiversity and the representativeness of a given sampling effort [64]. Curves were created for benthic and pelagic catches in each lake basin. In order to maximize sample size, and since there was no observed difference in catch composition of the two net types, the analysis included the full dataset with 408 nets in ULC and 99 nets in LLC.

*Precision*. To estimate the precision of abundance estimates, catches (NPUE) were log(x+1)-transformed, and the coefficient of variation of the mean (CV) was calculated according to Degerman et al. [31]: CV = SE/NPUE where SE is standard error and NPUE is mean NPUE. The calculation included data of all nets for total abundance in both benthic and pelagic zones, as well as separately for dominant benthic and pelagic fish species. The CEN standard uses the CV and the same calculation method as a measure of precision and requires the statistically ensured detection of a 50% difference in abundance of dominant species between two sampling occasions. It is equivalent to a CV of 0.1 or lower. As our sampling design is primarily developed for monitoring in the context of the WFD, this specification was used setting the minimum required sample size. Dominant species were defined as those comprising at least 30% of the catch in NPUE [26, 31].

### Calculation of prospective sample size

Our approach, with reduced net numbers deployed to sample large lakes, is based on the identification of distinct benthic fish communities at different depths. The analyses were carried out on the entire dataset (CEN and MOD) for the benthic zone. We used canonical analysis of principal coordinate (CAP, PRIMER ® 7, PRIMER-E Ltd, Ivybridge) ordination to visualize clusters of fish communities [65] over a Bray-Curtis similarity matrix computed with a dummy value of 1, using log(x+1)-transformed species-specific NPUE values. Following the visual inspection of the CAP ordination plot, we separated the depth strata into new classes: 0–11.9 m, 12–19.9 m, 20–49.9 m (LLC) and 0–19.9 m, 20–34.9 m, 35–49.9 m, 50–250 m (ULC). We tested for significant differences in fish species composition and abundance among the new depth strata using an ANOSIM test with 999 permutations (PRIMER ® 7, PRIMER-E Ltd, Ivybridge).

The number of nets required to sample each depth-dependent fish community was calculated according to the formula of Pringle [43]:

$$Number\;of\;nets=\frac{{\left(SD\right)}^{2}}{{\left(NPUE\right)}^{2}*{\left(CV\right)}^{2}}$$

We also used an alternative approach based on a power t-test, in which we computed the number of nets needed to detect a 50% difference in the mean NPUE of the dominant fish species in the respective depth-dependent fish communities with a power of 0.8 and significance level of 0.05. Calculation was made in the TOSTER package [55] in R [56]. When a fish community was found to extend across multiple depth strata, the number of nets calculated by both approaches were distributed across the defined depth strata according to their proportion of the total volume. The number of nets required to detect 90% of species within a depth stratum was identified. Species richness curves were calculated for each depth stratum using the SPECCACUM function in the ‘vegan’ package of R software. The minimum net requirement for a particular depth stratum was defined as the larger of the two values calculated, to ensure accurate estimate of dominant species abundance and detection of 90% of all species.

## Results

### Characteristics of fish communities

In ULC (408 nets), 9212 specimens were caught comprising 27 species, reduced to 25 by grouping whitefish (S1 Table in Supporting information). In LLC, 99 nets caught 1983 specimens of 19 species. In both basins, benthic and pelagic catches were heavily dominated by a single species. In the benthic zone of ULC, European perch *Perca fluviatilis* L. contributed 76% of total NPUE and, in LLC, 89% (S2, S3 Tables in Supporting information). In both basins three-spined stickleback *Gasterosteus aculeatus* L. was the dominant species of the pelagic zone, with 88% of total NPUE in ULC and 63% in LLC. Similarly, large proportions of biomass were distributed among few species. Perch held the largest share in the benthic zones of both basins, while whitefish *Coregonus* spp. dominated the pelagic zone.

### Comparison of CEN and MOD nets

#### Total catch

Using the MOD nets resulted in a significant reduction in fish catches compared to the CEN nets in both ULC and LLC. In ULC, MOD replicates captured 42% fewer specimens (2063 versus 3541), while in LLC, the reduction was 54% (360 versus 776). Overall, the use of MOD replicates led to a 48% reduction in fish mortality for the entire lake. The net-area adjusted NPUE showed comparable numbers. The NPUE of the MOD nets was 9.0% lower at ULC and 14.5% lower at LLC compared to the CEN nets (Tables 3, 4). The NPUE of benthic MOD nets was lower in 50% of the area-reduced panels (mesh sizes 6.25–12.5). The greater area of large mesh sizes in benthic MOD nets did not increase the NPUE. The comparison of pelagic CEN and MOD nets showed similar patterns in NPUE for the area-reduced panels.

**Table 3:** Abundance, biomass, and net area corrected values for NPUE (individuals per 100 m^2^ net area) and BPUE (biomass per 100 m^2^ net area), mean total catches and standard error (SE) of CEN and MOD nets in ULC according to mesh size.

		CEN						MOD					
Zone	Mesh size [mm]	Total catch [n]	NPUE [n/100 m^2^]	Mean [n]	SE	Total biomass [g]	BPUE [g/100 m^2^]	Total catch [n]	NPUE [n/100 m^2^]	Mean [n]	SE	Total biomass [g]	BPUE [g/100 m^2^]
***Benthic zone***	6.25	113	3013	1.40	0.57	510	13,592	69	3680	0.85	0.31	321	17,104
	8	249	6640	3.07	0.81	1189	31,701	134	7147	1.65	0.38	914	48,731
	10	1194	31,840	14.74	2.53	8688	231,680	495	26,400	6.11	1.22	3659	195,131
	12.5	1082	28,853	13.36	2.09	13,628	363,400	485	25,867	5.99	1.19	5568	296,949
	15.5	307	8187	3.79	0.66	7721	20,5893	273	7280	3.37	0.59	6419	171,163
	19.5	164	4373	2.02	0.36	6441	171,763	190	5067	2.35	0.45	8047	214,579
	24	74	1973	0.91	0.20	6807	181,523	68	1813	0.84	0.16	5305	141,453
	29	27	720	0.33	0.08	5216	139,091	53	707	0.65	0.18	8778	117,033
	35	21	560	0.26	0.07	4890	130,389	66	440	0.83	0.25	18,861	125,739
	43	12	320	0.15	0.05	6633	176,869	47	157	0.60	0.14	25,117	83,723
***Pelagic zone***	6.25	28	187	1.08	0.56	63	421	7	93	0.27	0.16	12	153
	8	151	1007	5.81	2.03	479	3193	46	613	1.77	0.52	152	2029
	10	94	627	3.62	1.04	367	2446	79	1053	3.04	1.04	289	3849
	12.5	9	60	0.35	0.18	63	421	1	13	0.04	0.04	11	147
	15.5	3	20	0.12	0.12	17	111	2	13	0.08	0.05	149	993
	19.5	1	7	0.04	0.04	53	353	1	7	0.04	0.04	39	260
	24	0	0	0.00	0.00	0	0	4	27	0.15	0.07	619	4,125
	29	9	60	0.35	0.12	1936	12,903	31	103	1.19	0.26	6030	20,101
	35	1	7	0.04	0.04	248	1653	10	17	0.38	0.17	2618	4363
	43	2	13	0.08	0.08	380	2533	2	2	0.08	0.05	1313	1094
Total		3541	88,467			65,327	1,669,937	2063	80,498			94,218	1,448,721

**Table 4:** Abundance, biomass, and net area corrected values for NPUE (individuals per 100 m^2^ net area) and BPUE (biomass per 100 m^2^ net area), mean total catches and standard error (SE) of CEN and MOD nets in LLC according to mesh size.

		CEN						MOD					
Zone	Mesh size [mm]	Total catch [n]	NPUE [n/100 m^2^]	Mean [n]	SE	Total biomass [g]	BPUE [g/100 m^2^]	Total catch [n]	NPUE [n/100 m^2^]	Mean [n]	SE	Total biomass [g]	BPUE [g/100 m^2^]
***Benthic zone***	6.25	29	773	1.45	0.39	54	1432	15	800	0.75	0.34	36	1909
	8	118	3147	5.90	3.54	744	19,832	54	2880	2.70	1.50	323	17,232
	10	185	4933	9.30	2.58	1236	32,955	149	7947	7.45	2.36	1089	58,101
	12.5	120	3200	6.05	2.11	1691	45,083	56	2987	2.80	0.75	742	39,563
	15.5	204	5440	10.20	9.00	2410	64,259	31	827	1.55	0.58	963	25,685
	19.5	27	720	1.35	0.90	1248	33,280	19	507	0.95	0.47	847	22,581
	24	8	213	0.45	0.27	975	26,008	9	240	0.45	0.27	789	21,037
	29	2	53	0.10	0.07	348	9280	4	53	0.21	0.12	702	9364
	35	5	133	0.25	0.12	1652	44,040	2	13	0.10	0.07	952	6347
	43	2	53	0.10	0.07	1383	36,885	9	30	0.45	0.21	3682	12,274
***Pelagic zone***	6.25	58	387	6.44	5.59	65	432	3	40	0.33	0.24	404	5380
	8	3	20	0.33	0.24	28	189	2	27	0.22	0.15	6	76
	10	11	73	1.22	0.66	49	329	0	0	0.00	0.00	0	0
	12.5	0	0	0.00	0.00	0	0	3	40	0.33	0.17	54	713
	15.5	0	0	0.00	0.00	0	0	0	0	0.00	0.00	0	0
	19.5	3	20	0.33	0.17	216	1437	0	0	0.00	0.00	0	0
	24	0	0	0.00	0.00	0	0	0	0	0.00	0.00	0	0
	29	1	7	0.11	0.11	315	2099	1	3	0.11	0.11	206	687
	35	0	0	0.00	0.00	0	0	1	2	0.11	0.11	346	577
	43	0	0	0.00	0.00	0	0	2	2	0.22	0.15	710	592
Total		776	19,173			12,413	317,541	360	16,397			11,850	222,118

#### Species richness and proportions

Despite the decrease in total catch, assessment of species richness was not hampered in MOD nets. In contrast, in ULC, MOD nets caught 24 species, two more than captured by CEN nets, and in LLC, MOD nets captured 16 species, one more than CEN. The additional species caught in ULC were lacustrine brown trout *Salmo trutta* L. and Prussian carp *Carassius gibelio* (Bloch 1782). In LLC, Prussian carp, white bream (L.), and stone loach *Barbatula barbatula* (L.) were only recorded in CEN nets, and chub *Squalius cephalus* (L.), common carp *Cyprinus carpio* L., burbot *Lota lota* (L.), and wels catfish *Silurus glanis* L. were caught only in MOD nets. The direct comparison of mesh-size catches of the net types showed a slight decrease in mean number of species in area-reduced panels of the MOD nets (mesh sizes 6.25–12.5 mm) while a minor increase was observed in area extended panels (mesh sizes 29–43 mm). The maximum difference was 0.3 species (mean difference ± SD for area-reduced panels: ULC = 0.13±0.06, LLC = 0.21±0.13; and for area extended panels: ULC = 0.21±0.11, LLC = 0.13±0.09). Equivalence tests with a range of 50% of the mean difference indicated no relevant differences in species numbers between the net types caught in mesh sizes from 6.25 mm to 12.5 mm in benthic sampling (S4A+S4C Table in Supporting information). The results were less clear for increased-area panels. The analysis in ULC showed no significant effect (S4A Table in Supporting information) while in LLC we cannot exclude an effect, as the 90% confidence interval was not within the defined equivalence range (S4C Table in Supporting information). In the pelagic zone, results were not clear for either the reduced or the extended panel sizes (S4B+S4D Table in Supporting information). Even in the unchanged panel sizes (mesh sizes 15.5–24.0 mm), the absence of a significant effect could not be excluded with certainty. The power of the analysis was high for most comparisons in the benthic zone of ULC. In contrast, power in the pelagic zone was low, except for comparisons with nets modified for 8 mm and 10 mm mesh. In LLC, the power obtained was generally poor.

In both lake basins, we did not find a significant effect on the number of species for the net type and the interaction between net type and the depth stratum in the GLMM model (ULC n = 2142, AICc = 8984.75; LLC n = 580, AICc 2686.19, Table 5). The explanatory factors of depth stratum, zone, and mesh size showed a significant effect on the species richness found in both lake basins (Table 5).

**Table 5:** Tests for the fixed effects in the GLMM models for the number of species and the log of the NPUE + 0.1 in Upper Lake Constance and Lower Lake Constance. In bold are factors with significant effects.

Variable	Effect	N parameters	DF denominator	F-value	p-value	
**Number of**	***Upper Lake Constance***					
**species**	Net type	1	658.8	0.443	0.506	
**(Poisson)**	**Depth stratum**	**7**	**197.6**	**17.254**	**< .0001**	*
	**Zone**	**1**	**224.9**	**38.450**	**< .0001**	*
	**Mesh size**	**1**	**2124**	**52.788**	**< .0001**	*
	Net type*depth stratum	7	203.1	0.212	0.982	
	***Lower Lake Constance***					
	Net type	1	201.4	0.145	0.704	
	**Depth stratum**	**5**	**24.2**	**9.376**	**< .0001**	*
	**Zone**	**1**	**62.6**	**11.750**	**0.001**	*
	**Mesh size**	**1**	**566**	**37.547**	**< .0001**	*
	Net type*depth stratum	5	45.9	0.421	0.832	
**Log(NPUE + .1)**	***Upper Lake Constance***					
**(Gaussian)**	Net type	1	185.4	0.030	0.864	
	**Depth stratum**	**7**	**174.7**	**20.374**	**< .0001**	*
	**Zone**	**1**	**187.7**	**56.745**	**< .0001**	*
	**Mesh size**	**1**	**1925.6**	**193.763**	**< .0001**	*
	Net type*depth stratum	7	185.2	0.246	0.973	
	***Lower Lake Constance***					
	Net type	1	43.6	0.709	0.404	
	**Depth stratum**	**5**	**41.1**	**10.723**	**< .0001**	*
	**Zone**	**1**	**33.7**	**13.174**	**0.001**	*
	**Mesh size**	**1**	**521**	**78.307**	**< .0001**	*
	Net type*depth stratum	5	43.5	0.092	0.993	

The 5 mm mesh, which was only present in CEN nets, caught four fish in ULC [two stone loach and two ruffe *Gymnocephalus cernua* (L.)] and in LLC (two stickleback, one stone loach, and one tench). No fish were caught in the 55 mm mesh.

**Fig 3:**
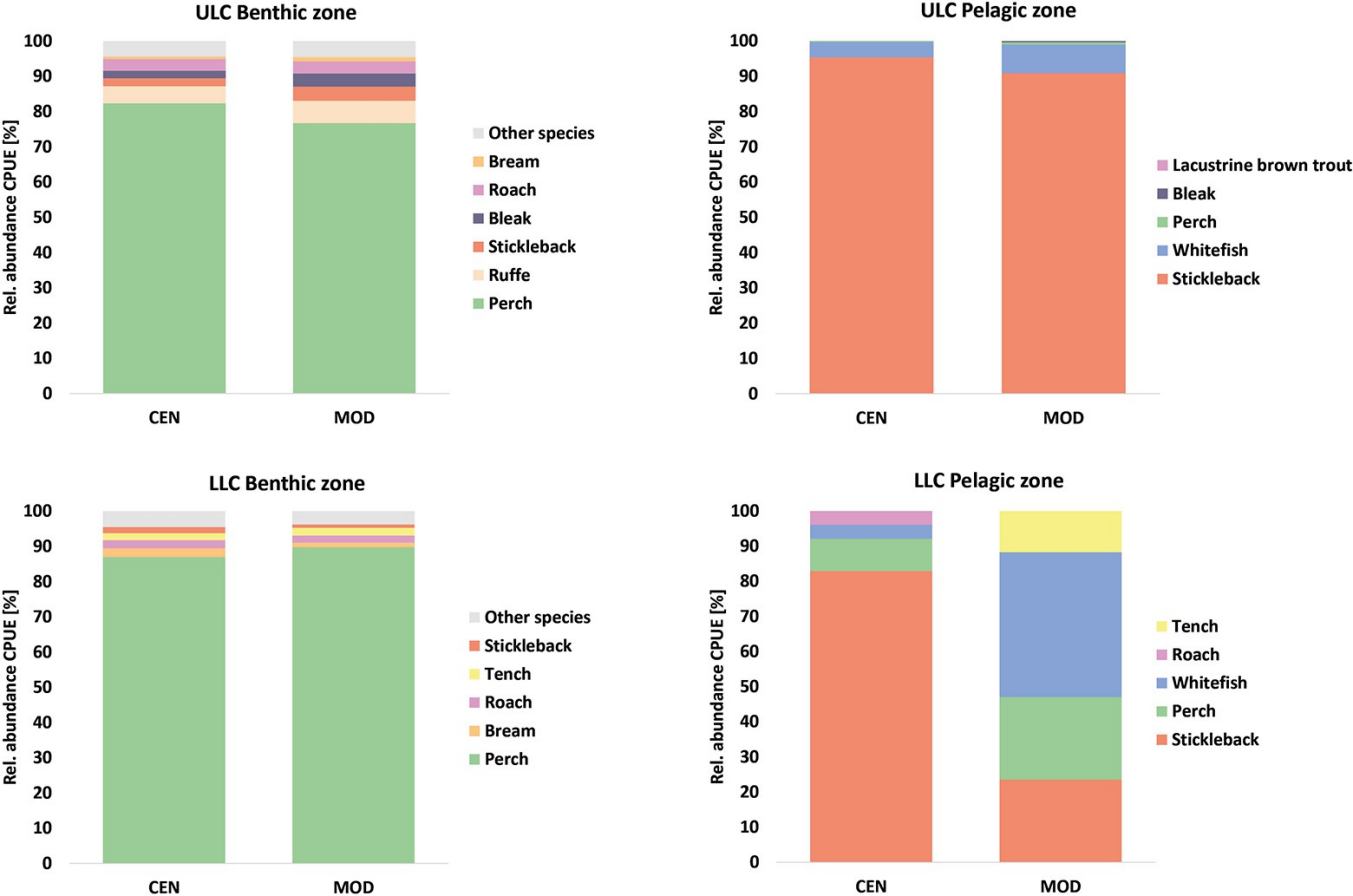
Proportions of species (NPUE) in CEN and MOD nets of the benthic and pelagic zone in ULC and LLC.

The net types revealed similar species structure, more pronounced in the benthic zone (Fig 3). The dominant species (>30%) showed differences of <10% (S5 Table in Supporting information). In the LLC pelagic zone, species proportions differed with net type. A single stickleback was documented in each of two MOD nets. One to four specimens were caught in three of the four CEN nets, with a mass catch of 56 stickleback in a single CEN net.

#### NPUE

The mean NPUE of small mesh sizes in MOD nets (area-reduced panels) was equivalent to CEN nets (S6 Table in Supporting information). A clear tendency of one net type to show higher NPUE was not observed. The equivalence tests revealed no meaningful effect of mesh size 6.25–12.5 mm in the benthic nets of either lake basin. In ULC, no effect was observed for mesh sizes 29–43 mm (S6A Table in Supporting information), while a minor effect in LLC is possible (S6C Table in Supporting information). In the pelagic zone, no unambiguous results were observed in either the reduced and extended panel sizes (S6B+S6D Table in Supporting information). Even within the panels of unchanged areas (mesh sizes 15.5–24.0), the absence of an effect could not be ruled out with certainty. Power was sufficient for most comparisons in the benthic zone of ULC. However, it was weak in the pelagic zone and in the LLC.

Similar to number of species, we found no significant effect on the log of the NPUE + 0.1 for the net type or the interaction between net type and depth stratum in the mixed effect model, in either of the basins (ULC n = 2142, AICc = 6771.54; LLC n = 580, AICc 1797.77, Table 5). The factors depth stratum, zone, and mesh size also showed a positive significant effect on the NPUE in both lake basins (Table 5).

#### Comparison of catch composition

**Fig 4:**
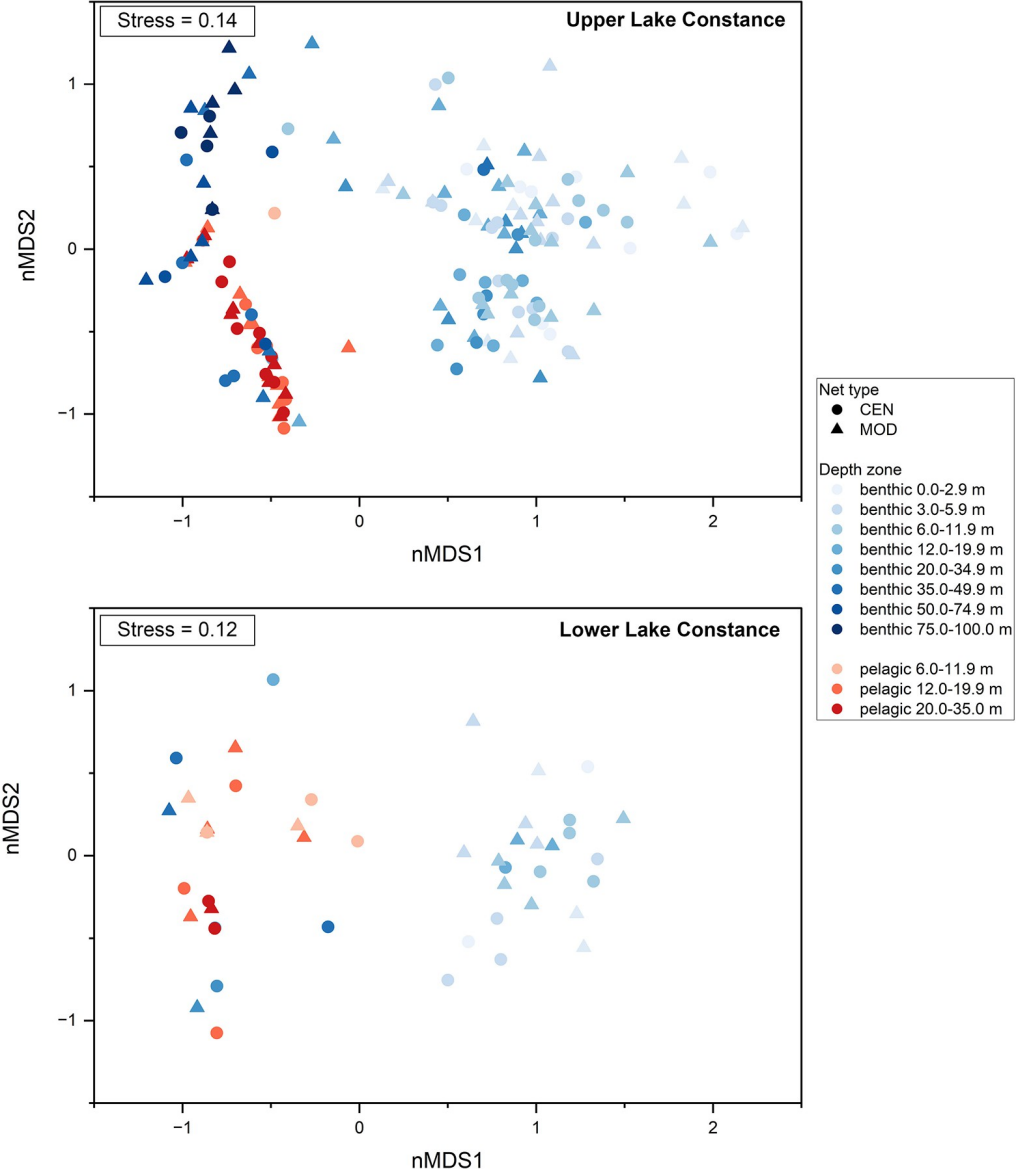
NMDS Plot of species-specific NPUE of CEN and MOD nets in ULC and LLC according to depth stratum and zone.

The nMDS analysis of ULC defined three distinct species communities associated with benthic samples to a depth of 34.9 m, benthic samples from deep strata (35–99.9 m), and pelagic samples (Fig 4). The CEN and MOD replicates were frequently in proximity and yielded similar catch compositions. Results of the PERMDISP showed differences in homogeneity of variance of CEN and MOD nets with respect to depth strata (PERMDISP, F = 4.304, P (perm) = 0.001) (S7 Table, S1 Fig in Supporting information). Significant differences were found among depth strata but not between CEN and MOD nets in the same depth stratum.

In LLC, samples from shallow benthic nets showed clusters at a depth of 19.9 m. Deeper benthic samples aggregated with pelagic samples in another area of the plot. Replicates of CEN and MOD nets generally grouped together or at least formed part of the same cluster (Fig 4). PERMDISP did not show differences in the variances of groups (PERMDISP, F = 2.9021, P(perm) = 0.204), suggesting that samples did not differ with zone, depth stratum, or net type (S7 Table, S1 Fig in Supporting information).

Since samples from both lake basins showed different benthic and pelagic species composition according to both nMDS plots and homogeneity of variance when using zone as a single group factor (PERMDISP, ULC: F = 242.74, P(perm) = 0.001, N = 214; LLC: F = 47.171, P(perm) = 0.001, N = 57), ANOSIM analysis was conducted separately for benthic and pelagic samples (S8 Table in Supporting information). Differences in benthic species communities among depth strata were common (ANOSIM, ULC: R-statistic = 0.427, p < 0.01; LLC: R-statistic = 0.398, p < 0.01), whereas fish communities of pelagic zones were more homogeneous, showing no significant differences with depth (ANOSIM, ULC: R-statistic = 0.042, p = 0.143; LLC: R-statistic = 0.01, p = 0.518). Catches of the CEN and MOD nets from the same depth stratum did not exhibit significant differences.

#### Work effort

The catch of the panels in the MOD nets that were half the size of those in the CEN nets in the benthic zone of the ULC was about 50% that of the CEN nets (S9A Table in Supporting information). Similar mesh/panel sizes produced nearly identical catches. Increased-area panels in the MOD nets yielded higher catches in ULC. In the LLC, a similar trend was evident in the benthic zone, but catches were lower (S9B Table in Supporting information). Since the catch in the pelagic zone of both lake basins was low, labour savings were calculated only for the benthic zones. With labour savings of 37% for ULC and 39% for LLC compared to CEN nets, MOD nets showed comparable savings for operations directly affected by fish catch (S9C+S9D Table in Supporting information).

### Utility of the sampling campaign

#### Species richness

**Fig 5:**
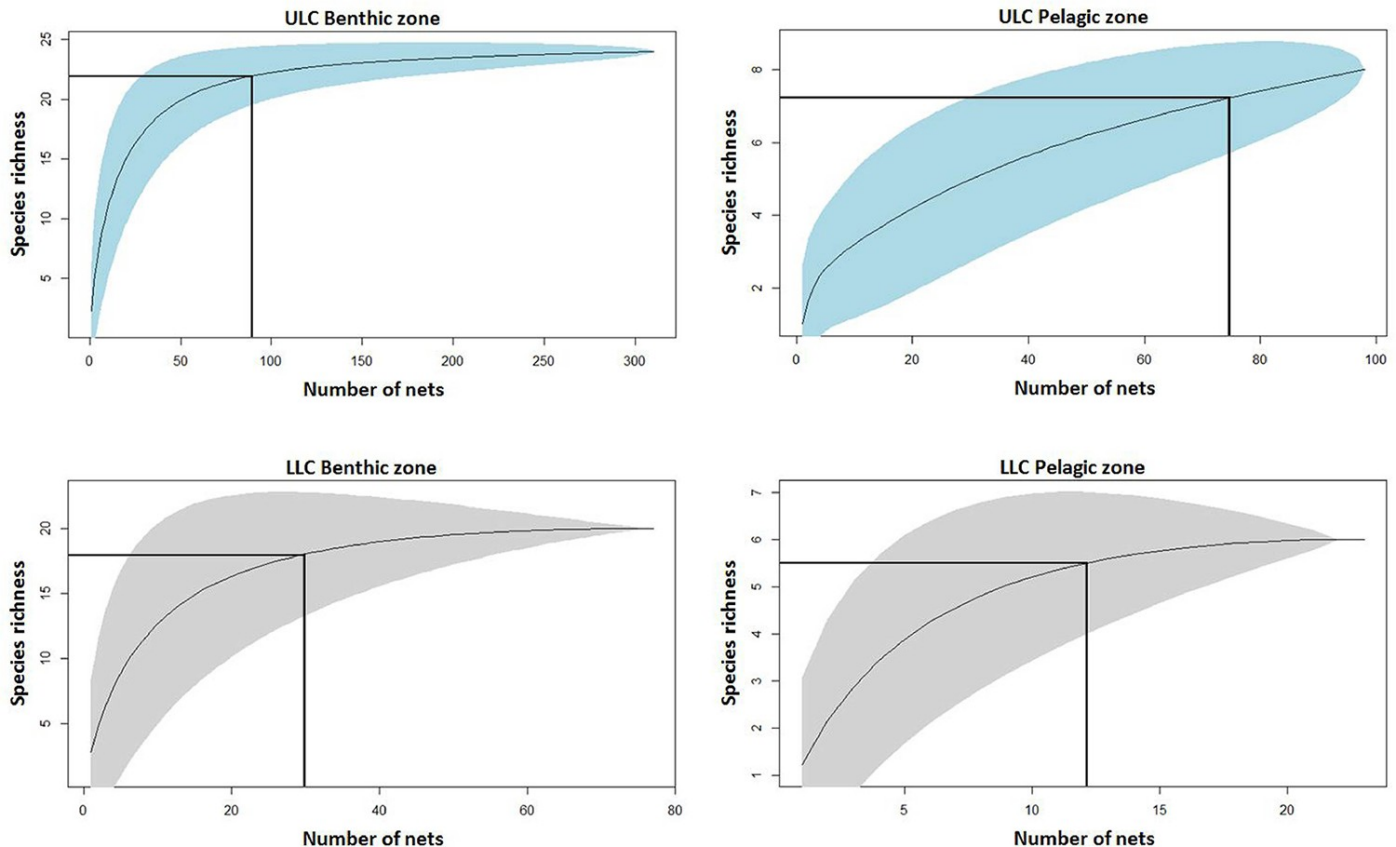
Species accumulation curves with 95% confidence intervals (shaded areas) in the benthic and pelagic zones of ULC and LLC. Curves represent expected species richness and lines show net numbers required for catch of 90% of the documented species with a probability of 50%.

The curves for benthic species richness of both basins displayed an acute rise with sampling effort before levelling to a well-defined plateau (Fig 5). Pelagic sampling in LLC showed a similar pattern, but in the ULC lacked the plateau phase, reflecting the occasional capture of rare species. In ULC, detection of 90% of documented pelagic species with a probability of 50% required 76% (n = 76) of deployed nets, compared to just 29% (n = 90) in the benthic zone. In LLC, 90% of the observed species richness was detected from 52% (n = 12) of pelagic nets and 39% (n = 30) of benthic nets.

### Precision of NPUE according to DIN 14757

The CV of benthic total abundance in both lake basins was less than 0.10. In the pelagic zone, the CV was 0.10 in ULC and 0.19 in LLC (Table 6). Perch was present in benthic samples of both lake basins, and stickleback was clearly dominant in pelagic samples, with no other species contributing more than 30% of abundance. The CV of perch was less than 0.10 in both lake basins, and therefore met the requirements of the CEN protocol. The CV of stickleback in the pelagic zone exceeded 0.10.

**Table 6:** Mean NPUE (log(x+1) transformed) of all deployed nets, independent of type, with standard deviation (SD) and coefficient of variation (CV) for total abundance and dominant species in the benthic and pelagic zones of ULC and LLC.

Lake basin	Waterbody zone	Total catch	Mean NPUE (SD)	CV	Dominating species	Mean NPUE (SD)	CV
Upper Lake Constance	Benthic		1.34 (±0.95)	0.04	Perch	1.06 (±1.02)	0.05
	Pelagic		0.48 (±0.48)	0.10	Stickleback	0.39 (±0.49)	0.13
Lower Lake Constance	Benthic		1.37 (±0.89)	0.07	Perch	1.18 (±0.96)	0.09
	Pelagic		0.39 (±0.35)	0.19	Stickleback	0.39 (±0.72)	0.39

### Calculation of a prospective sample size

**Fig 6:**
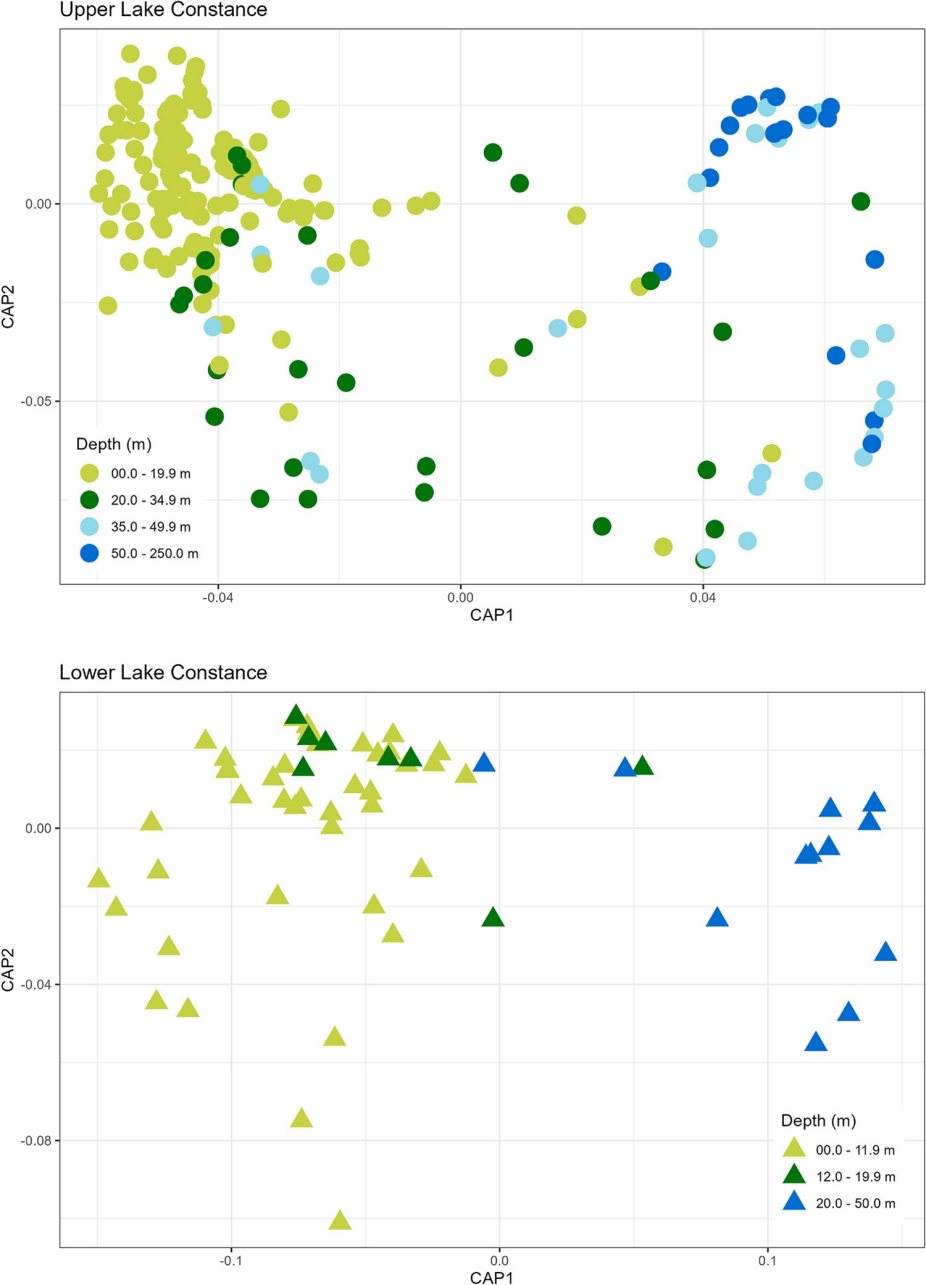
Depth stratum–related CAP chart using a Bray-Curtis similarity matrix of log(x+1) transformed species-specific NPUE values for benthic samples of ULC and LLC.

The CAP plot for ULC showed shallow (0–19.9 m) and deep (50–250 m) samples to form clearly separated clusters, with samples from 20–34.9 m and 35–49.9 m occupying a transitional region (Fig 6). The ANOSIM identified significant differences in benthic fish communities of different depth strata in ULC (ANOSIM, R-statistic = 0.41, p = 0.001) (S10 Table in Supporting information). Samples from the 20–49.9 m and 50–250 m strata were distinct from those of the 0–19.9 m layer. However, in pairwise comparison of depth strata, the communities of 20–49.9 m strata were not distinguishable from those at lower depth. Since the majority of samples from 20–34.9 m grouped closer to samples from 0–19.9 m, and the majority of samples from 35–49.9 m grouped closer to 50–250 m, the transitional strata were treated as distinct, resulting in four identifiable benthic fish communities.

The CAP plot for LLC showed a strong overlap of depth zones 0–11.9 and 12–19.9 m, allowing those strata to be pooled for the subsequent net number calculations (Fig 6). Samples from deep strata (20–50 m) clustered separately in the ordination space. The ANOSIM for LLC also revealed significant differences among depth strata (ANOSIM, R-statistic = 0.442, p = 0.001), with three distinguishable fish communities in the pairwise comparison of depth strata (S10 Table in Supporting information). The first group included samples from 0–11.9 m and the second those from 20–50 m. ANOSIM results did not distinguish samples from 12–19.9 m from either shallower or deeper strata, and they can consequently be considered transitional.

The focus on depth-related fish communities allowed for a significant reduction in the number of nets required to reliably estimate abundance of dominant species using the formula of Pringle [65]. To 34.9 m, the number of nets needed to record 90% of detected species within a depth stratum was greater than the minimum for abundance estimates of dominant species in ULC (Table 7). Fish densities of strata deeper than 50 m in ULC and 20 m in LLC were too low for accurate abundance estimates to be made with reasonable fishing effort. The minimum number of benthic nets required to detect at least 90% of all species with a probability of 50% and adequately estimate abundance of dominant species at depths to 49.9 m in ULC is 170. In LLC, 42 benthic nets are required to achieve the same accuracy to a depth of 19.9 m. The power-based approach for the calculation of net numbers considering the NPUE representation generally provided comparable results.

**Table 7:** Minimum net requirements for a prospective sampling campaign according to the DIN 14757 calculation in ULC and LLC. Net numbers are calculated based on depth-dependent fish communities (ULC four communities, LLC two communities) of the present sampling campaign. The calculated net numbers ensure accurate estimate of the NPUE of the dominant species and detection of 90% of species present with 50% probability within depth strata. Calculated net numbers for NPUE estimate using a power-based approach to ensure a power (β) of 0.8 and significance level α = 0.05 are shown.

Depth stratum [m]	Min. net number species		Min. net number NPUE			Min. total net number for the sampling campaign
	Species richness	Number of nets	Dominating species	DIN 14757	Power-based approach	
***Upper Lake Constance***						
0–2.9	23	30.2	Perch	7.8	3.9	31 (30.2)
3–5.9		12.9		3.3	1.7	13 (12.9)
6–11.9		11.5		3.0	1.5	12 (11.5)
12–19.9		11.4		2.9	1.5	12 (11.4)
20–34.9	7	26.0	Perch	22.6	79.7	26 (26.0)
35–49.9	7	17.0	Perch	24.3	352.4	25 (24.3)
50–74.9	7	8.0	Arctic char	139.4	81.6	8 (8.0)
75–99.9		6.9		120.0	70.2	7 (6.9)
100–149.9		15.9		276.8	161.9	16 (15.9)
150–199.9		10.9		189.9	111.1	11 (10.9)
200–250		8.4		145.9	85.4	9 (8.4)
**Total**		**159.1**		**936**	**950.7**	**170 (166.4)**
***Lower Lake Constance***						
0–2.9	17	9.3	Perch	9.7	5.3	10 (9.7)
3–5.9		3.3		3.5	1.9	4 (3.5)
6–11.9		2.6		2.7	1.4	3 (2.7)
12–19.9		7.9		8.2	4.5	9 (8.2)
20–34.9	4	12.9	Perch	691.6	380.3	13 (12.9)
35–50		2.1		110.7	60.9	3 (2.1)
**Total**		**38**		**826.4**	**454.2**	**42 (38)**

Apart from reducing the number of nets required for the detection of 90% of the species detected with a probability of 50% (74 nets ULC, 12 nets LLC), net numbers for pelagic samples could not be further reduced, as precision of the abundance of the dominant species was already larger than recommended by the CEN protocol (Table 6). Determination of depth-dependent fish communities was not possible, as pelagic samples did not indicate differences among strata (S8B+S8D Table in Supporting information). Therefore, variation could not be reduced by generating depth-dependent subgroups.

## Discussion

The standardized CEN gillnet fishing protocol, originally developed for sampling lake fish communities in northern Europe, has become a popular tool for the investigation of fish communities in a variety of natural [66] and artificial lakes [11] throughout Europe, despite being invasive and highly labour intensive in large lakes [37]. The present study describes a modified net type and limited sampling design capable of significantly reducing both fish mortality and work effort without sacrificing information.

### Application of MOD nets

The results of the study show that deployment of MOD nets reduces both fish mortality and labour per net. Although the direct comparison of mesh sizes points to a marginal reduction in species detection in area-reduced panels of the small mesh sizes, the total observed species richness was greater when using MOD nets. Species caught in only one net type showed low abundance and included rare species and difficult-to-net species. The reduction in panel sizes of benthic nets showed no meaningful effect on number of documented species or the NPUE. The absence of a relevant difference was also demonstrated for mesh sizes with extended panel sizes in the benthic zone of ULC. However, for the LLC benthic zone a difference was observed. Relevant differences in species numbers and/or NPUE could not be analysed with certainty in net type comparisons of the entire pelagic zone. Comparisons with a risk of relevant difference were only seen in instances of low catch numbers and, rarely, low net numbers. Thus, results of the benthic net catches represent the most reliable results. The comparisons in which differences were observed were likely the result of the small number of fish in these zones and the patchy distribution of large individuals than to the different net types.

Despite the lower catch numbers, the biomass extracted by MOD nets was greater than that of the CEN nets in the ULC. This was due to the higher catch in the area increased panels (29–43 mm mesh sizes) in the MOD nets. The overall low catch numbers, however, meant that the increase had no significant impact on the work effort. A threat to the population resulting from the removal of large spawners is also unlikely, as the nets covered an extremely small area of the lake.

Size selectivity is a general limitation in gillnet sampling, with small fish being frequently underrepresented compared to large fish [67–69], but, as NPUE values in mesh sizes 6.25–12.5 mm did not show a meaningful difference between net types, this effect was not detected. Electrofishing is generally a more appropriate technique for sampling small size classes in fish community monitoring and, in combination with multi-mesh gillnets, is the recommended procedure for evaluation of the ecological status of lakes within the WFD [70].

The catch of mesh sizes present only in CEN nets did not provide information relevant to fish community. In the 55 mm segments, no fish were caught, and the 5 mm segments captured only eight specimens, none belonging to a species or year class that was not present in the next larger mesh size. Only the mean total length of two stone loach (5.8±0.35 cm) caught in the 5 mm mesh in ULC was slightly less than that of those captured in the next larger mesh size of 6.25 mm (7.1±0.56, N = 32). The low catch, with reduced fish mortality and work effort, may be a weak argument for eliminating those mesh sizes. However, combined with the little information derived their removal is justified. The oligotrophic character of Lake Constance and the related comparatively slow growth of young-of-the-year fish [71], in combination with spring spawning (for most species) and summer sampling, make it possible that juvenile fish in the more productive lakes of Central Europe are large enough to be caught in the 6.25 mm mesh size. Even in lakes with many large fish, where calculation of species richness and abundance is important, the lack of information concerning these small fish is acceptable, but comparability to CEN assessments might be reduced. The generally low catches and limited consequences with respect to mortality and labour, might justify use of 55 mm and larger mesh in MOD nets in those lakes.

Despite the lower total catches in MOD nets, the dominance structure of the most common species was similar in the net types. This is particularly important for lake assessment for the WFD using the German standard software DeLFI [42]. Minimizing the ecological impact of data collection should be a guiding principle in any scientific or conservation monitoring program, and the ability of MOD nets to limit mortality without losing relevant information is a considerable advantage. Some European countries, including the U.K., Ireland, Belgium, and the Netherlands, limit the use of gillnets because of their invasive nature [19, 72]. Reduction in work effort, particularly in the time- and labour-intensive processing of the catch, reduces costs and maximizes funding of sampling campaigns. While our calculation of 38% reduction on average effort achieved in Lake Constance is substantial, it is potentially greater in more productive lakes when scaled to a campaign covering hundreds of sampling sites.

### Appropriateness of sampling effort

#### Species richness

Compared to CEN standards, the smaller number of nets used in the present study reduced labour and fish mortality while delivering adequate representation of species richness in both lake basins, with the exception of the pelagic zone of ULC in which species richness estimates continued to increase with sampling effort. All primarily pelagic species known to be currently present in the lake were documented, including whitefish, arctic char, and stickleback as well as the rare lacustrine brown trout. The lack of a plateau was predominantly related to the occasional capture of benthic or littoral species such as perch, roach *Rutilus rutilus* (L.) and bleak *Alburnus alburnus* (L.) in pelagic nets. The sampling campaign detected 20 of the 26 native fish species listed by Eckmann & Rösch [48] as inhabiting ULC before 1900 (coregonids counted as a single species), plus five additional species not present in the original inventory. Sampling of LLC yielded 16 of the 27 species listed as native by Zaugg et al. [73], plus three non-native species. Species or forms not captured in either basin during the current study include the extremely rare grayling *Thymallus thymallus* (L.), common nase *Chondrostoma nasus* (L.), barbel *Barbus barbus* (L.), European bitterling *Rhodeus amarus* (Bloch 1782), sunbleak *Leucaspius delineatus* (Heckel 1843), gudgeon *Gobio gobio* L., Eurasian minnow *Phoxinus phoxinus* (L.), the extinct bottom whitefish *Coregonus gutturosus* (Gmelin 1818), and the European eel *Anguilla anguilla* (L.), which is only rarely recorded in gillnet catches [34].

Additional information about the extant littoral fish community was obtained from a simultaneous campaign of electrofishing, which yielded small numbers of European eel, barbel, gudgeon, Crucian carp *Carassius carassius* (L.), along with bitterling in ULC and European eel and barbel in LLC. With the exception of the European eel, the results confirm these species as minority populations in Lake Constance, but nevertheless suggest that the study achieved near-complete documentation of all species currently present. The European eel must be considered separately, as Lake Constance holds a substantial population, of which ~10,000 mature each year [74]. There is a general view that electrofishing underestimates benthic species [75], particularly those whose occurrence is strongly related to the availability of adequate shelter or to their occupancy of deep-water habitats, as is the case with eel [76, 77]. As a further confounding factor, eels tend to be easily immobilized by electrofishing and to consequently remain in shelter [78]. Several species that appeared only in electrofishing samples were caught close to in- or out-flows. In lakes connected to moving water, riverine species can make a significant contribution to species diversity [79]. Those species, primarily present at inflows, are often rheoparous, e.g., barbel and gudgeon [80], or prefer rheophilic conditions during at least one developmental stage, and are thus considered river mouth or ‘guest’ species in Lake Constance. They tend to be rarely recorded, and are often absent from surveys depending on the fishing technique used. Nevertheless, absence of such species might also indicate ecological disruption, for example to habitat connectivity [81]. Thus, it is important that inflow and river sections immediately above and below lakes be included in evaluation of ecological conditions.

The absence from gillnets of some species caught by electrofishing can be related to more general characteristics. The passive nature of gillnets leads to selectivity [34], and species with shape, size, or behaviour that makes them hard to catch (e.g., barbel, bitterling, eel, gudgeon, sunbleak) are gillnetted so rarely that using this method alone to record all species would require unfeasibly intense fishing effort [31, 68]. Since the species caught solely by electrofishing were missing from both net types, their absence from MOD nets can be considered a general limitation of gillnet sampling. Nevertheless, it serves to emphasize the value of complementary methods to generate a comprehensive picture of the fish community [19, 70, 82].

The lower number of nets used in this study, in combination with littoral electrofishing, appear to have provided adequate representation of species diversity. The question remains of whether routine ecological status monitoring needs to include capture of species known to be rare [18, 83]. The abundance of indicator species might be sufficient to reveal ecological status, making records of scarce species with similar ecological demands redundant. In the case of scarce species relying on specific ecological niches or possessing complex life cycles sensitive to habitat characteristics, specific monitoring for presence or even precise estimates of abundance might be advisable. Although the inventory of species detected via gillnetting in this study was not complete, several comparatively rare species were recorded, including stone moroko *Pseudorasbora parva* (Temminck & Schlegel 1846), pumpkinseed *Lepomis gibbosus* (L.), and bullhead *Cottus gobio* L.. These species make little if any contribution to the ecological assessment of the waterbody, and most were documented in the simultaneous electrofishing campaign. The prospective sampling design developed here for Lake Constance was required to detect 90% species known to be present. This was obtained by fewer nets, reducing both work effort and fish mortality. When necessary, additional monitoring for scarce species might be accomplished in a less resource-intensive and destructive manner, such as long line fishing, beach seining, scuba diving, or snorkelling [83].

#### Abundance precision

In terms of abundance sampling, precision can be defined as the coefficient of variation of the mean [26]. In lake systems, it is usually linked to population density and is correlated to trophic status [84]. Despite exhibiting lower phosphorus levels, ULC yielded more precise sampling of dominant species in both benthic and pelagic zone, at CV of 5% and 13%, respectively. The corresponding CV values in benthic and pelagic zones of LLC were 9% and 39%. Estimates of dominant benthic zone species were relatively accurate in both lake basins. For comparison, Deceliere-Vergès et al. [18] investigated the precision of their sampling campaign of 14 natural French lakes, following the CEN protocol. As the lakes were smaller than Lake Constance, they used fewer nets (mean = 27±21) than in our study, which can lead to higher CV values. Despite the differing sample sizes, their CV values are discussed to show examples of the precision achieved by the CEN protocol. They obtained mean CV values of 20±18% for roach and 26±25% for perch in benthic samples. Phosphorus concentrations in the lakes ranged from 5 to 131 μg *L*^-1^ but exceeded 20 μg *L*^-1^ in only two. In 26 Scandinavian lakes with phosphorous concentrations from 7 to 20 μg *L*^-1^, Holmgren et al. [34] found median CV for total benthic abundance of ~25% using a similar sampling protocol and calculation method. We found total benthic abundance CV of 4% in ULC and 7% in LLC. Given that the lakes in the cited studies exhibited higher phosphorus levels than found in Lake Constance, precision should be better than that obtained in the present study. An explanation for the exceptionally high precision of sampling in Lake Constance is the dominance of perch, with relative abundance averaging 83% and consistent distribution across vast areas of the benthic zone, in combination with the large sample size. The proportion of the second most abundant species was <30% in both lake basins, a value at which they were not considered as dominant and therefore had no impact on the benthic sample size.

Precision of the NPUE was poor for deep strata in the benthic zone. In ULC, only seven species were caught in benthic strata below 50 m, and precision decreased dramatically because of low fish density. In the shallower LLC, similar patterns were detected at just 20 m depth, beyond which sampling revealed only four species and a clear decline in fish density. Obtaining an accurate estimate of actual relative abundance in those deep areas would require unfeasibly large samples, and since such areas generally show low species numbers and fish density [18, 85, 86], a reduction in sampling effort may be seen as acceptable in fish-based evaluation of lakes.

The monitoring of Lake Constance provides an ecological overview rather than a detailed assessment of deep-water fish communities. However, lake deep-water habitats do not always have low fish density, and some may possess a considerable proportion of total abundance or biomass [40]. It is desirable to obtain records of rare and sensitive deep-water species, and, in Lake Constance, this is particularly true of the endemic deep-water char [87, 88], rediscovered in 2014 and likely to be vulnerable to the effects of climate change. The increased thermal stratification caused by rising water temperatures can impede water turnover [4] and exacerbate the risk of hypoxia in deep zones, posing a clear threat to this temperature- and oxygen-sensitive salmonid [89, 90]. Hence, verification of specialized deep-water species might be relevant to the assessment of entire lake conditions, with those species serving as indicators of integrated effects of climate change over time. The minimum net numbers proposed here take into account the value of recording a complete species inventory of deep benthic strata, while accepting the limitations of gillnetting in benthic settings [82].

Our results for pelagic sampling also provide valuable information on whole-lake fish communities, but since the precision of pelagic abundance has not, to our knowledge, been reported for any previous campaign, it is not possible to make comparisons. The CV of total abundance in the present study was 10% in ULC and 19% in LLC, with that of dominant species greater. Despite low pelagic fish density, total precision exceeded the average benthic precision reported by Holmgren [34] in Scandinavian lakes and is considered adequate for pelagic sampling in this context.

### Calculation of prospective sampling size and accordance with WFD standards

The method used to calculate net numbers based on fish communities identified in the lake can be considered a habitat-specific assessment approach in which the depth zones (ULC 0–19.9 m, 20–34.9 m, 35–49.9 m, 50–74.9 m; LLC 0–19.9 m, 20–50 m) are considered habitats with different fish communities. Both approaches for the calculation of net numbers ([65] or power-based) for abundance estimate recommended considerably fewer nets than the random and stratified calculation method of the CEN protocol [26], which is based on lake morphology. Although the power-based net number was usually lower in individual depth zones, the DIN 14757 calculation formula based on Pringle [43] is still recommended for monitoring water bodies. While the CEN protocol would result in 685 nets for Lake Constance, only 212 nets were needed with the method based on the fish communities in combination with the formula of Pringle [43].

The CEN protocol, designed to meet requirements of the WFD, demands an abundance sampling precision of 10% (CV) for dominant benthic species [26]. This was met by the benthic net numbers applied in our campaign. The requirements were not met by our sampling of deep benthic strata or the pelagic zone and will not be achieved by the prospective sampling design, as the required sampling effort exceeds feasibility. For example, obtaining a CV of 10% for the dominant pelagic species, stickleback, would require deployment of 157 nets in ULC and 554 in LLC, an effort entirely disproportionate to information yielded that would result in high fish mortality. In both pelagic and deep benthic zones of Lake Constance, the challenge for sampling lies in sparse fish populations and sample sizes too small for accurate estimates of dominant species abundance. For those areas, the sampling design proposed here must be focused on obtaining a complete species inventory.

### Limitations of the study

The results presented here are drawn from a single fish-sampling campaign in the two lake basins during which the observed distribution and composition of the fish community will have been shaped by specific biotic and abiotic factors existing at that time [91]. Since interannual variations in fish populations will influence the precision of any sampling effort, the suggested net numbers will require verification by further campaigns. Although this study treated the two lake basins of Lake Constance as distinct, the small sample size and limited species variety mean that the results are not sufficiently robust to transfer to other large lakes. Factors such as trophic status, species composition and activity, size, and shape of fish will all have an impact on catch composition [25], and the applicability of MOD nets must be demonstrated in other lake contexts.

Savings in labour apply only to Lake Constance. Notwithstanding the need for further investigation, the concept of reducing sampling effort and fish mortality by modifying an existing sampling design to focus on depth-dependent fish communities is probably transferable, as long as distinct fish communities can be recognized and sampling precision is taken into account.

### Limitations of gillnet sampling

Beyond the invasive, laborious, and expensive nature of gillnet sampling, limitations include its dependency on fish activity, differences in the catchability of species [92], underestimation of small size classes [69], and the fact that catch data indicate only relative abundance [27, 28, 93]. The efficacy of the method can be influenced by mesh congestion, declining as the number of fish trapped increases [94, 95]. During daytime in clear water, even small numbers of entangled fish can reduce further catch significantly [25]. In alpine lakes with pristine water, this effect should be anticipated, and fishing conducted when light levels are low. As net fishing in this study was performed from dusk till dawn in late summer and early autumn, light levels and visibility were minimal, and the CPUE could consequently only be improved by shortening the duration of net deployment. However, the need to cover both nycthemeral migration periods would then require a midnight net change.

The standard European CEN protocol indicates that the sampling method provides abundance estimates only for fish between 40 mm to 400 mm. Estimates of some less catchable species (e.g. European eel, burbot, bullhead and pike *Esox Lucius* L.), as well as small fish (0+ and 1+), may be underestimated. These facts must be taken into account during assessments [26]. Another important factor that affects gillnet selectivity is the girth. However, multi-mesh nets based on a geometric series of mesh sizes, like the nets used in our study, minimize this factor because adjacent mesh sizes cover each other [20, 96].

### Alternative sampling methods

The search for methods to replace gillnet sampling is ongoing, but all currently existing alternatives are subject to some form of bias [17]. In practice, gillnet sampling remains the method of choice, supplemented with other techniques to obtain a complete picture of community structure. Hydroacoustic surveying is non-invasive, appropriate for total abundance and biomass assessments of pelagic fish species [97]. But despite progress in species identification using wideband acoustics [98], it is not yet possible to isolate and identify single species within complex pelagic communities, and the technique is still less effective in benthic or shallow habitats [99].

Another relatively new and promising approach in biodiversity monitoring is the analysis of environmental DNA (eDNA). Sampling is cost-effective [100, 101] and completely non-invasive, as the eDNA is extracted from water samples [102]. The detection capacity of the technique is high, and even in large or difficult-to-sample habitats it can provide accurate biodiversity assessment including proof of rare species [103–105]. Some eDNA studies have achieved qualitative and quantitative descriptions of entire fish communities [106–108]. Hänfling et al. [109] used eDNA metabarcoding to describe the fish communities of three large deep lakes in the UK. However, while eDNA provides information regarding species relative abundance, further study is needed in diverse ecosystems to characterize relationships of abundance and compare those observed, for example, in quantitative PCR analyses [110]. It will also be important to understand the factors influencing the quantity, distribution, and aggregation of DNA in the environment, and within a specific lake (e.g., spawning seasons, lake mixis, and aggregation of DNA along the shoreline) [99, 111]. Even if eDNA sampling does supersede conventional gillnet sampling at some point, the ecological assessment of lakes demands information it cannot provide, for example concerning population size and age structure and physical condition (e.g., nutritional condition, diseases, parasites) of the species present, hence the need for supplementary data from conventional sampling methods will remain.

A further cost-effective way to obtain data regarding fish stock is from commercial fisheries or citizen science sources such as recreational fisheries [112]. However, this type of sampling has limitations, as procedures are not standardized, identification is not performed by professionally trained staff, and data from commercial and recreational fisheries tends to cover specific target species of a certain size class and is thus inadequate for the multi-species assessment required to evaluate the ecological condition of a waterbody. These potential biases are diverse and often poorly understood and further exacerbate the challenge of data validation [113–115].

## Conclusions

The novel net type used in this study can significantly reduce sampling effort and fish mortality without loss of information. In addition, a new depth-based sampling design for ecological assessment of fish communities as required by the WFD reduces the number of nets providing species coverage and precision comparable to that of the existing CEN protocol in shallower benthic habitats. The catch data provide less precise data in deep benthic and pelagic habitats and would limit the technique in those areas to the documentation of species. Nevertheless, the monitoring covers all metrics relevant to the WFD for evaluating the fish community of a large deep lake and therefore offers a promising approach to improve the efficiency of multi-mesh gillnet monitoring programs. The reduction of fish mortality, labour requirements and overall ecological impacts should increase stakeholder and general public acceptance of such monitoring programs. These results must be verified with data from other large lakes before the method can be applied more widely in the assessment of large lake fish communities.

## Supporting information

S1 Table Total catch in Upper and Lower Lake Constance. (PDF)

S2 Table Combined catch per unit effort of Upper Lake Constance. (PDF)

S3 Table Combined catch per unit effort of Lower Lake Constance. (PDF)

S4 Table Differences of species numbers between CEN and MOD nets. (PDF)

S5 Table Catch per unit effort of species with >1% of Upper and Lower Lake Constance. (PDF)

S6 Table Differences of NPUE between CEN and MOD nets. (PDF)

S7 Table Test of homogeneity of variances in Upper and Lower Lake Constance. (PDF)

S8 Table ANOSIM analysis of CEN and MOD nets of each depth stratum. (PDF)

S9 Table Calculation of labour savings using MOD nets. (PDF)

S10 Table ANOSIM analysis of NPUE data of benthic nets of Upper and Lower Lake Constance. (PDF)

S1 Fig Variation in species composition and number of individuals of depth strata in Upper and Lower Lake Constance. (PDF)
