# Supplementary material for: Obtaining accurate population estimates with reduced workload and lower fish mortality in multi-mesh gillnet sampling of a large pre-alpine lake
Source: PLoS One. 2024 Mar 18;19(3):e0299774. doi: 10.1371/journal.pone.0299774 (PMC10947718; doi:10.1371/journal.pone.0299774)
Supplement: S1 Table — (PDF) [file pone.0299774.s001.pdf]

Table S1. Total catch of all CEN and MOD nets in Upper Lake Constance (ULC) and Lower Lake Constance (LLC).

Species	Scientific name	ULC	LLC	Status
European perch	*Perca fluviatilis*	6272	1636	native
Three-spined stickleback	*Gasterosteus aculeatus*	846	97	non-native
Ruffe	*Gymnocephalus cernua*	573	7	non-native
Roach	*Rutilus rutilus*	520	55	native
Bleak	*Alburnus alburnus*	223	16	native
Whitefish	*Coregonus spp.*	164	21	native
White bream	*Blicca bjoerkna*	134	3	native
Freshwater bream	*Abramis brama*	94	31	native
Chub	*Squalius cephalus*	68	6	native
Common dace	*Leuciscus leuciscus*	61	3	native
Rudd	*Scardinius erythrophthalmus*	61	10	native
Pikeperch	*Sander lucioperca*	43	16	non-native
Stone loach	*Barbatula barbatula*	40	4	native
Pike	*Esox lucius*	21	29	native
Burbot	*Lota lota*	20	1	native
Arctic char	*Salvelinus umbla*	18	-	native
Tench	*Tinca tinca*	12	31	native
Wels catfish	*Silurus glanis*	11	8	native
Deepwater char	*Salvelinus profundus*	9	-	native
Prussian carp	*Carassius gibelio*	7	1	native
Common carp	*Cyprinus carpio*	6	8	native
Bullhead	*Cottus gobio*	4	-	native
Pumpkinseed	*Lepomis gibbosus*	3	-	non-native
Stone moroko	*Pseudorasbora parva*	1	-	non-native
Brown trout	*Salmo trutta*	1	-	native
