# Supplementary material for: Obtaining accurate population estimates with reduced workload and lower fish mortality in multi-mesh gillnet sampling of a large pre-alpine lake
Source: PLoS One. 2024 Mar 18;19(3):e0299774. doi: 10.1371/journal.pone.0299774 (PMC10947718; doi:10.1371/journal.pone.0299774)
Supplement: S2 Table — (PDF) [file pone.0299774.s002.pdf]

Table S2. Combined catch per unit effort of all CEN and MOD nets including all depth strata in number and biomass and associated values for species proportion (Prop), mean (per 100 m^2 net area), maximum (Max) and standard deviation (SD) of species caught in benthic and pelagic nets of Upper Lake Constance.

	NPUE [n/100 m^2]						BPUE [g/100 m^2]					
Species	Total	Prop [%]	Mean	Median	Max	SD	Total	Prop [%]	Mean	Median	Max	SD
Benthic zone												
European perch	23,140.4	76.2	94.8	37.3	982.0	137.3	299,598.1	56.2	1,227.9	495.2	10,319.2	1718.3
Ruffe	1801.9	5.9	7.4	0.0	184.7	22.7	39,891.8	7.5	163.5	0.0	5526.1	522.1
Three-spined stickleback	1760.4	5.8	7.2	0.0	765.3	50.8	6675.7	1.3	27.4	0.0	3084.5	204.0
Roach	1275.7	4.2	5.2	0.0	211.3	17.2	61,427.5	11.5	251.8	0.0	10,042.1	841.7
Bleak	866.2	2.9	3.6	0.0	101.3	13.2	6936.7	1.3	28.4	0.0	803.1	109.9
Freshwater bream	368.4	1.2	1.5	0.0	90.7	9.3	3996.8	0.7	16.4	0.0	1296.7	103.9
White bream	212.0	0.7	0.9	0.0	44.4	4.4	18,744.8	3.5	76.8	0.0	3500.0	405.0
Common dace	171.3	0.6	0.7	0.0	29.3	3.4	7630.0	1.4	31.3	0.0	1005.3	137.2
Stone loach	144.9	0.5	0.6	0.0	33.3	3.1	423.5	0.1	1.7	0.0	75.6	7.8
Rudd	136.2	0.4	0.6	0.0	72.0	6.1	5445.4	1.0	22.3	0.0	2910.9	219.8
Whitefish	133.4	0.4	0.5	0.0	12.0	1.6	22,217.6	4.2	91.1	0.0	2053.3	266.5
Chub	94.8	0.3	0.4	0.0	23.0	2.0	16,388.1	3.1	67.2	0.0	2389.3	285.2
Pikeperch	46.4	0.2	0.2	0.0	6.7	0.8	17,020.9	3.2	69.8	0.0	1649.7	255.4
Lake char	39.9	0.1	0.2	0.0	5.7	0.8	4173.3	0.8	17.1	0.0	746.7	86.5
Burbot	38.9	0.1	0.2	0.0	5.3	0.7	5370.9	1.0	22.0	0.0	778.7	90.7
Tench	34.1	0.1	0.1	0.0	10.7	0.9	499.7	0.1	2.0	0.0	231.0	17.3
Deepwater char	24.9	0.1	0.1	0.0	5.3	0.6	698.9	0.1	2.9	0.0	176.3	18.7
Wels catfish	19.4	0.1	0.1	0.0	6.7	0.6	4203.6	0.8	17.2	0.0	1226.7	106.5
Bullhead	18.2	0.1	0.1	0.0	5.3	0.6	77.6	0.0	0.3	0.0	32.0	2.8
Pike	12.8	0.0	0.1	0.0	2.2	0.3	10,082.4	1.9	41.3	0.0	1948.9	209.9
Common carp	6.6	0.0	0.0	0.0	2.7	0.2	1112.8	0.2	4.6	0.0	750.0	50.2
Stone moroko	5.3	0.0	0.0	0.0	5.3	0.3	12.8	0.0	0.1	0.0	12.8	0.8
Prussian carp	4.7	0.0	0.0	0.0	2.7	0.2	415.5	0.1	1.7	0.0	65.7	10.0
Pumpkinseed	4.2	0.0	0.0	0.0	2.2	0.2	179.0	0.0	0.7	0.0	91.4	8.1
Pelagic zone												
Three-spined stickleback	420.5	87.7	6.1	1.8	44.8	9.6	1353.4	16.3	19.6	5.9	157.7	31.9
Whitefish	31.8	6.6	0.5	0.3	3.0	0.6	6351.7	76.3	92.1	50.0	575.3	120.3
Bleak	20.0	4.2	0.3	0.0	7.3	1.3	286.7	3.4	4.2	0.0	157.3	21.4
European perch	3.9	0.8	0.1	0.0	1.3	0.2	47.0	0.6	0.7	0.0	14.7	2.8
Deepwater char	1.3	0.3	0.0	0.0	1.3	0.2	19.7	0.2	0.3	0.0	19.7	2.4
Lake char	1.0	0.2	0.0	0.0	0.7	0.1	159.0	1.9	2.3	0.0	82.8	13.4
Roach	0.7	0.1	0.0	0.0	0.7	0.1	30.3	0.4	0.4	0.0	30.3	3.7
Brown trout	0.1	0.0	0.0	0.0	0.1	0.0	77.8	0.9	1.1	0.0	77.8	9.4
