# Supplementary material for: Obtaining accurate population estimates with reduced workload and lower fish mortality in multi-mesh gillnet sampling of a large pre-alpine lake
Source: PLoS One. 2024 Mar 18;19(3):e0299774. doi: 10.1371/journal.pone.0299774 (PMC10947718; doi:10.1371/journal.pone.0299774)
Supplement: S3 Table — (PDF) [file pone.0299774.s003.pdf]

Table S3. Combined catch per unit effort of all CEN an MOD nets including all depth strata in number and biomass and associated values for species proportions (Prop), mean (per 100 m^2 net area), maximum (Max) and standard deviation (SD) of species caught in benthic and pelagic nets of Lower Lake Constance.

	NPUE [n/100 m^2]						BPUE [g/100 m^2]					
**Species**	**Total**	**Prop [%]**	**Mean**	**Median**	**Max**	**SD**	**Total**	**Prop [%]**	**Mean**	**Median**	**Max**	**SD**
Benthic zone												
European perch	6217.3	88.7	101.9	55.6	1144.0	185.5	69,087.2	63.8	1,132.6	541.3	11,045.9	2009.6
Roach	164.2	2.3	2.7	0.0	32.0	5.2	4463.6	4.1	73.2	0.0	819.7	166.8
Three-spined stickleback	133.3	1.9	2.2	0.0	85.3	11.1	140.4	0.1	2.3	0.0	92.8	12.1
Freshwater bream	109.3	1.6	1.8	0.0	17.8	4.3	554.1	0.5	9.1	0.0	120.5	25.1
Tench	90.4	1.3	1.5	0.0	21.3	3.6	1460.9	1.3	23.9	0.0	519.3	79.9
Pike	65.6	0.9	1.1	0.0	16.7	2.7	11,129.7	10.3	182.5	0.0	2734.0	485.6
Bleak	59.1	0.8	1.0	0.0	21.3	3.1	176.5	0.2	2.9	0.0	59.7	9.1
Pikeperch	31.9	0.5	0.5	0.0	8.9	1.5	7820.9	7.2	128.2	0.0	2,701.3	426.3
Ruffe	25.3	0.4	0.4	0.0	5.3	1.3	321.2	0.3	5.3	0.0	85.9	18.1
Stone loach	18.2	0.3	0.3	0.0	16.0	2.1	48.5	0.0	0.8	0.0	45.9	5.9
Rudd	17.3	0.2	0.3	0.0	6.0	1.0	3585.2	3.3	58.8	0.0	2004.2	294.1
Wels catfish	16.3	0.2	0.3	0.0	6.0	1.0	3969.1	3.7	65.1	0.0	3112.0	399.8
Common carp	16.3	0.2	0.3	0.0	12.0	1.6	636.9	0.6	10.4	0.0	299.5	43.7
Whitefish	13.8	0.2	0.2	0.0	4.4	0.8	2207.7	2.0	36.2	0.0	1211.1	163.9
White bream	12.9	0.2	0.2	0.0	5.3	1.0	63.2	0.1	1.0	0.0	44.8	5.9
Common dace	7.1	0.1	0.1	0.0	4.4	0.7	608.7	0.6	10.0	0.0	394.0	57.0
Chub	5.7	0.1	0.1	0.0	3.0	0.5	1509.3	1.4	24.7	0.0	689.7	114.2
Prussian carp	7.5	0.1	0.1	0.0	5.3	1.9	386.7	0.5	5.4	0.0	298.7	231.2
Burbot	1.3	0.0	0.0	0.0	1.3	0.2	235.6	0.2	3.9	0.0	235.6	30.2
Pelagic zone												
Three-spined stickleback	40.8	62.7	2.3	0.0	33.9	7.9	47.1	3.5	2.6	0.0	38.5	9.0
Whitefish	7.6	11.6	0.4	0.1	1.3	0.5	1135.0	84.8	63.1	18.6	534.7	130.3
European perch	6.9	10.6	0.4	0.0	3.6	0.9	52.6	3.9	2.9	0.0	24.5	7.0
Roach	4.5	6.9	0.2	0.0	1.3	0.5	96.5	7.2	5.4	0.0	53.4	13.8
Tench	2.7	4.1	0.1	0.0	1.3	0.4	4.4	0.3	0.2	0.0	3.1	0.8
Bleak	2.7	4.1	0.1	0.0	1.3	0.4	3.5	0.3	0.2	0.0	1.9	0.6
