# Supplementary material for: Obtaining accurate population estimates with reduced workload and lower fish mortality in multi-mesh gillnet sampling of a large pre-alpine lake
Source: PLoS One. 2024 Mar 18;19(3):e0299774. doi: 10.1371/journal.pone.0299774 (PMC10947718; doi:10.1371/journal.pone.0299774)
Supplement: S4 Table — (PDF) [file pone.0299774.s004.pdf]

Table S4. Number of nets (N), log mean, log standard deviation (SD), log standard error (SE), 95 % confidence interval, mean and SD for number of species caught in each mesh size in CEN and MOD nets and difference between net types. For an equivalence test +/-20% and +/-50% of the mean difference we report the equivalence bound (Eq. margin 20%/50%) and the result of the test (-ΔL < 90 CI < ΔU, Test 20%/50%). For a significance level of alpha = 0.05, we also report the obtained power for the current sample size and the number of nets to achieve a power of 0.8. For each mesh size, data of depth levels were pooled for CEN and MOD net comparison.

A) Benthic zone of Upper Lake Constance.

		MESH SIZE [mm]									
		**6.25**	**8**	**10**	**12.5**	**15.5**	**19.5**	**24**	**29**	**35**	**43**
**CEN**	N	81	81	81	81	81	81	81	81	81	81
	log mean	0.3457	0.6790	0.9259	0.7037	0.7531	0.7160	0.4444	0.2716	0.1852	0.1358
	log SD	0.6738	0.7878	1.0097	0.7322	0.9815	0.9385	0.7071	0.5247	0.3909	0.4403
	log SE	0.0749	0.0875	0.1122	0.0814	0.1091	0.1043	0.0786	0.0583	0.0434	0.0489
	log 95 % CI	0.2 – 0.49	0.51 – 0.85	0.71 – 1.15	0.54 – 0.86	0.54 – 0.97	0.51 – 0.92	0.29 – 0.6	0.16 – 0.39	0.1 – 0.27	0.04 – 0.23
	mean	0.346	0.679	0.926	0.704	0.753	0.716	0.444	0.272	0.185	0.136
	SD	0.674	0.788	1.009	0.732	0.981	0.938	0.707	0.525	0.391	0.44
**MOD**	N	81	81	81	81	81	81	81	81	81	81
	log mean	0.2222	0.5062	0.6667	0.6543	0.7407	0.7037	0.5062	0.4074	0.4691	0.3827
	log SD	0.4743	0.7267	0.8660	0.7609	0.9718	0.9804	0.7093	0.8182	0.8956	0.7995
	log SE	0.0527	0.0807	0.0962	0.0845	0.1080	0.1089	0.0788	0.0909	0.0995	0.0888
	log 95 % CI	0.12 – 0.33	0.35 – 0.66	0.48 – 0.86	0.49 – 0.82	0.53 – 0.95	0.49 – 0.92	0.35 – 0.66	0.23 – 0.59	0.27 – 0.66	0.21 – 0.56
	mean	0.222	0.506	0.667	0.654	0.741	0.703	0.506	0.107	0.469	0.383
	SD	0.474	0.727	0.866	0.761	0.971	0.98	0.709	0.818	0.895	0.799
**Diff.**	mean diff	0.123	0.173	0.259	0.049	0.012	0.012	-0.062	-0.136	-0.284	-0.247
	log SD	0.544	0.551	0.653	0.475	0.652	0.672	0.608	0.721	0.833	0.692
	90 % CI	0.091	0.118	0.147	0.116	0.151	0.148	0.110	0.107	0.109	0.102
	ratio mean	0.642	0.745	0.720	0.929	0.984	0.982	1.140	0.393	2.535	2.816
Eq. margin 20 %		0.057	0.119	0.159	0.136	0.149	0.142	0.095	0.068	0.065	0.052
Eq. margin 50 %		0.142	0.296	0.398	0.340	0.373	0.355	0.238	0.170	0.164	0.130
Test 20 %		FALSE	TRUE	TRUE	TRUE	FALSE	FALSE	FALSE	FALSE	FALSE	FALSE
Test 50 %		**TRUE**	**TRUE**	**TRUE**	**TRUE**	TRUE	TRUE	TRUE	**TRUE**	**TRUE**	**TRUE**
**Power**											
Eq. margin 20 %		0.00	0.00	0.01	0.13	0.00	0.00	0.00	0.00	0.00	0.00
Eq. margin 50 %		0.03	0.92	0.97	1.00	0.95	0.91	0.60	0.00	0.00	0.00
**Number of nets**											
Eq. margin 20 %		1570.94	371.37	288.51	209.93	326.88	384.10	700.90	1930.35	2778.82	3052.20
Eq. margin 50 %		251.92	60.00	46.75	34.18	52.88	62.04	112.72	309.43	445.18	488.92

B) Pelagic zone of Upper Lake Constance.

		MESH SIZE [mm]									
		**6.25**	**8**	**10**	**12.5**	**15.5**	**19.5**	**24**	**29**	**35**	**43**
	N	26	26	26	26	26	26	26	26	26	26
	log mean	0.2692	0.5769	0.6154	0.1538	0.0385	0.0385	0.0000	0.2692	0.0385	0.0385
	log SD	0.4523	0.5038	0.4961	0.3679	0.1961	0.1961	0.0000	0.4523	0.1961	0.1961
	log SE	0.0887	0.0988	0.0973	0.0722	0.0385	0.0385	0.0000	0.0887	0.0385	0.0385
	log 95 % CI	0.1 – 0.44	0.38 – 0.77	0.42 – 0.81	0.01 – 0.3	-0.04 – 0.11	-0.04 – 0.11	0 – 0	0.1 – 0.44	-0.04 – 0.11	-0.04 – 0.11
	mean	0.269	0.577	0.615	0.154	0.038	0.038	0	0.27	0.038	0.038
	SD	0.452	0.503	0.496	0.368	0.196	0.196	0	0.452	0.196	0.196
**MOD**	N	26	26	26	26	26	26	26	26	26	26
	log mean	0.1154	0.5000	0.5000	0.0385	0.0769	0.0385	0.1538	0.6154	0.1923	0.0769
	log SD	0.3258	0.5099	0.5099	0.1961	0.2717	0.1961	0.3679	0.4961	0.4019	0.2717
	log SE	0.0639	0.1000	0.1000	0.0385	0.0533	0.0385	0.0722	0.0973	0.0788	0.0533
	log 95 % CI	-0.01 – 0.24	0.3 – 0.7	0.3 – 0.7	-0.04 – 0.11	-0.03 – 0.18	-0.04 – 0.11	0.01 – 0.3	0.42 – 0.81	0.04 – 0.35	-0.03 – 0.18
	mean	0.115	0.5	0.5	0.038	0.077	0.038	0.154	0.615	0.192	0.077
	SD	0.326	0.51	0.509	0.197	0.272	0.196	0.368	0.496	0.402	0.272
**Diff.**	mean diff	0.154	0.077	0.115	0.115	-0.038	0.000	-0.154	-0.346	-0.154	-0.038
	log SD	0.430	0.471	0.402	0.326	0.326	0.272	0.368	0.510	0.430	0.326
	90 % CI	0.111	0.140	0.140	0.083	0.066	0.054	0.075	0.140	0.090	0.066
	ratio	0.428	0.867	0.813	0.247	2.026	1.000	-	2.278	5.053	2.026
Eq. margin 20 %		0.038	0.108	0.112	0.019	0.012	0.008	0.015	0.088	0.023	0.012
Eq. margin 50 %		0.096	0.269	0.279	0.048	0.029	0.019	0.038	0.221	0.058	0.029
Test 20 %		FALSE	FALSE	FALSE	FALSE	FALSE	FALSE	FALSE	FALSE	FALSE	FALSE
Test 50 %		**FALSE**	**TRUE**	**TRUE**	**FALSE**	FALSE	FALSE	FALSE	**TRUE**	**FALSE**	**FALSE**
**Power**											
Eq. margin 20 %		0.00	0.00	0.00	0.00	0.00	0.00	0.00	0.00	0.00	0.00
Eq. margin 50 %		0.00	0.30	0.59	0.00	0.00	0.00	0.00	0.02	0.00	0.00
**Number of nets**											
Eq. margin 20 %		2138.21	327.85	223.07	4917.01	13656.48	21375.36	9797.04	569.74	5937.60	13656.48
Eq. margin 50 %		342.68	53.04	36.28	787.29	2185.71	3420.73	1568.20	91.74	950.69	2185.71

C) Benthic zone of Lower Lake Constance.

		MESH SIZE [mm]									
		**6.25**	**8**	**10**	**12.5**	**15.5**	**19.5**	**24**	**29**	**35**	**43**
**CEN**	N	20	20	20	20	20	20	20	20	20	20
	log mean	0.45	0.6	0.95	0.9	0.6	0.45	0.25	0.1	0.25	0.1
	log SD	0.8870	0.6806	1.0501	0.9119	0.5982	0.8256	0.5501	0.3078	0.5501	0.3078
	log SE	0.1983	0.1522	0.2348	0.2039	0.1338	0.1846	0.1230	0.0688	0.1230	0.0688
	log 95 % CI	0.06 – 0.84	0.3 – 0.9	0.49 – 1.41	0.5 – 1.3	0.34 – 0.86	0.09 – 0.81	0.01 – 0.49	-0.03 – 0.23	0.01 – 0.49	-0.03 – 0.23
	mean	0.7	0.6	0.95	0.9	0.6	0.45	0.25	0.1	0.25	0.1
	SD	0.917	0.658	0.974	0.88	0.67	0.834	0.55	0.338	0.509	0.608
**MOD**	N	20	20	20	20	20	20	20	20	20	20
	log mean	0.7	0.4	0.75	0.75	0.75	0.5	0.2	0.15	0.1	0.35
	log SD	0.8013	0.6806	0.7864	0.7164	0.7864	0.7609	0.4104	0.3663	0.3078	0.6708
	log SE	0.1792	0.1522	0.1758	0.1602	0.1758	0.1701	0.0918	0.0819	0.0688	0.1500
	log 95 % CI	0.35 – 1.05	0.1 – 0.7	0.41 – 1.09	0.44 – 1.06	0.41 – 1.09	0.17 – 0.83	0.02 – 0.38	-0.01 – 0.31	-0.03 – 0.23	0.06 – 0.64
	mean	0.45	0.4	0.75	0.75	0.75	0.5	0.2	0.15	0.1	0.35
	SD	0.887	0.681	0.786	0.716	0.786	0.761	0.41	0.366	0.308	0.671
**Diff.**	mean diff	-0.250	0.200	0.200	0.150	-0.150	-0.050	0.050	-0.050	0.150	-0.250
	log SD	0.605	0.571	0.923	0.759	0.444	0.910	0.550	0.444	0.587	0.587
	90 % Ci	0.270	0.217	0.295	0.260	0.222	0.251	0.153	0.107	0.143	0.170
	ratio mean	0.643	0.667	0.789	0.833	1.250	1.111	0.800	1.500	0.400	3.500
Eq. margin 20 %		0.115	0.100	0.170	0.165	0.135	0.095	0.045	0.025	0.035	0.045
Eq. margin 50 %		0.288	0.250	0.425	0.413	0.338	0.238	0.113	0.063	0.088	0.113
Test 20 %		FALSE	FALSE	FALSE	FALSE	FALSE	FALSE	FALSE	FALSE	FALSE	FALSE
Test 50 %		**TRUE**	**TRUE**	**TRUE**	**TRUE**	TRUE	FALSE	FALSE	**FALSE**	**FALSE**	**FALSE**
**Power**											
Eq. margin 20 %		0.00	0.00	0.00	0.00	0.00	0.00	0.00	0.00	0.00	0.00
Eq. margin 50 %		0.01	0.00	0.01	0.08	0.53	0.00	0.00	0.00	0.00	0.00
**Number of nets**											
Eq. margin 20 %		474.41	559.58	505.99	363.25	186.17	1573.86	2560.37	5408.75	4820.72	2916.50
Eq. margin 50 %		76.48	90.11	81.54	58.70	30.38	252.39	410.23	866.08	771.88	467.21

D) Pelagic zone of Lower Lake Constance.

		MESH SIZE [mm]									
		**6.25**	**8**	**10**	**12.5**	**15.5**	**19.5**	**24**	**29**	**35**	**43**
**CEN**	N	9	9	9	9	9	9	9	9	9	9
	log mean	0.3333	0.2222	0.4444	0.0000	0.0000	0.3333	0.0000	0.1111	0.0000	0.0000
	log SD	0.7071	0.4410	0.7265	0.0000	0.0000	0.5000	0.0000	0.3333	0.0000	0.0000
	log SE	0.2357	0.1470	0.2422	0.0000	0.0000	0.1667	0.0000	0.1111	0.0000	0.0000
	log 95 % CI	-0.13 – 0.8	-0.07 – 0.51	-0.03 – 0.92	0 – 0	0 – 0	0.01 – 0.66	0 – 0	-0.11 – 0.33	0 – 0	0 – 0
	mean	0.444	0.222	0.444	0	0	0.333	0	0.111	0	0
	SD	0.527	0.441	0.726	0	0	0.5	0	0.333	0	0
**MOD**	N	9	9	9	9	9	9	9	9	9	9
	log mean	0.4444	0.2222	0.0000	0.3333	0.0000	0.0000	0.0000	0.1111	0.1111	0.2222
	log SD	0.5270	0.4410	0.0000	0.5000	0.0000	0.0000	0.0000	0.3333	0.3333	0.4410
	log SE	0.1757	0.1470	0.0000	0.1667	0.0000	0.0000	0.0000	0.1111	0.1111	0.1470
	log 95 % CI	0.1 – 0.79	-0.07 – 0.51	0 – 0	0.01 – 0.66	0 – 0	0 – 0	0 – 0	-0.11 – 0.33	-0.11 – 0.33	-0.07 – 0.51
	mean	0.333	0.222	0	0.333	0	0	0	0.111	0.111	0.222
	SD	0.707	0.441	0	0.5	0	0	0	0.333	0.333	0.441
**Diff.**	mean diff	-0.111	0.000	0.444	-0.333	0.000	0.333	0.000	0.000	-0.111	-0.222
	log SD	0.726	0.441	0.726	0.500	0.000	0.500	0.000	0.441	0.333	0.441
	90 % CI	0.302	0.213	0.273	0.191	0.000	0.191	0.000	0.161	0.117	0.161
	ratio mean	0.750	1.000	0.000	-	-	0.000	-	1.000	-	-
Eq. margin 20 %		0.078	0.044	0.044	0.033	0.000	0.033	0.000	0.022	0.011	0.022
Eq. margin 50 %		0.194	0.111	0.111	0.083	0.000	0.083	0.000	0.056	0.028	0.056
Test 20 %		FALSE	FALSE	FALSE	FALSE	FALSE	FALSE	FALSE	FALSE	FALSE	FALSE
Test 50 %		**FALSE**	**FALSE**	**FALSE**	**FALSE**	FALSE	FALSE	FALSE	**FALSE**	**FALSE**	**FALSE**
**Power**											
Eq. margin 20 %		0.00	0.00	0.00	0.00	-	0.00	-	0.00	0.00	0.00
Eq. margin 50 %		0.00	0.00	0.00	0.00	-	0.00	-	0.00	0.00	0.00
**Number of nets**											
Eq. margin 20 %		1494.98	1686.68	4576.98	3854.41	-	3854.41	-	6744.03	15414.93	6744.03
Eq. margin 50 %		239.77	270.44	732.89	617.27	-	617.27	-	1079.72	2467.06	1079.72
