# Supplementary material for: Obtaining accurate population estimates with reduced workload and lower fish mortality in multi-mesh gillnet sampling of a large pre-alpine lake
Source: PLoS One. 2024 Mar 18;19(3):e0299774. doi: 10.1371/journal.pone.0299774 (PMC10947718; doi:10.1371/journal.pone.0299774)
Supplement: S5 Table — (PDF) [file pone.0299774.s005.pdf]

Table S5. Catch per unit effort (NPUE) of species with >1 % in the benthic and pelagic zones of Upper Lake Constance and Lower Lake Constance.

**Benthic zone**						**Pelagic zone**					
	**Proportion**			**SE**			**Proportion**			**SE**	
**Species**	**CEN**	**MOD**	**Difference [%]**	**CEN**	**MOD**	**Species**	**CEN**	**MOD**	**Difference [%]**	**CEN**	**MOD**
Upper Lake Constance											
Perch	82	77	6.86	4.7	4.8	Stickleback	95	91	4.71	9.1	8.4
Ruffe	5	6	-31.43	2.0	1.6	Whitefish	4	8	-85.94	4.6	7.6
Stickleback	2	4	-75.49	2.5	2.2						
Bleak	2	4	-79.04	0.7	1.6						
Roach	3	4	-3.56	0.6	1.0						
Bream	1	1	-75.25	0.4	0.5						
Lower Lake Constance											
Perch	87	90	-3.24	8.9	9.9	Stickleback	83	24	71.62	16.1	11.8
Bream	3	1	48.76	5.2	2.9	Perch	9	234	-155.46	11.3	12.7
Roach	2	2.	11.73	0.9	0.9	Whitefish	4	41	-943.14	8.7	13.8
Tench	2	2	-9.12	0.9	1.3	Roach	4	0	100.00	8.7	-
Stickleback	2	1	42.35	7.1	4.6	Tench		12	-100.00	-	5.6
