# Supplementary material for: Obtaining accurate population estimates with reduced workload and lower fish mortality in multi-mesh gillnet sampling of a large pre-alpine lake
Source: PLoS One. 2024 Mar 18;19(3):e0299774. doi: 10.1371/journal.pone.0299774 (PMC10947718; doi:10.1371/journal.pone.0299774)
Supplement: S6 Table — (PDF) [file pone.0299774.s006.pdf]

Table S6. Number of nets (N), log mean, log standard deviation (SD), log standard error (SE), log 95 % confidence interval, mean and SD for NPUE of each mesh size of CEN and MOD nets and difference between net types. For an equivalence test +/-20% and +/-50% of the mean difference we report the equivalence bound (Eq. margin 20%/50%) and the result of the test (-ΔL < 90 CI < ΔU, Test 20%/50%). For a significance level of alpha = 0.05, we also report the obtained power for the current sample size and the number of nets required to achieve a power of 0.8. For each mesh size, data of depth levels were pooled for CEN and MOD net comparison.

A) Benthic zone of Upper Lake Constance.

		MESH SIZE [mm]									
		**6.25**	**8**	**10**	**12.5**	**15.5**	**19.5**	**24**	**29**	**35**	**43**
**CEN**	N	81	81	81	81	81	81	81	81	81	81
	log mean	0.4910	0.9563	1.4790	1.4385	1.0128	0.8864	0.5901	0.3636	0.2873	0.1569
	log SD	0.8452	1.0278	1.3389	1.3489	1.1253	1.0027	0.8553	0.6662	0.6102	0.4791
	log SE	0.0939	0.1142	0.1488	0.1499	0.1250	0.1114	0.0950	0.0740	0.0678	0.0532
	log 95 % CI	0.31 – 0.68	0.73 – 1.18	1.19 – 1.77	1.14 – 1.73	0.77 – 1.26	0.67 – 1.1	0.4 – 0.78	0.22 – 0.51	0.15 – 0.42	0.05 – 0.26
	mean	37.202	81.975	393.086	356.214	101.070	53.992	24.362	8.889	6.914	3.951
	SD	135.691	194.172	606.055	501.948	151.731	99.063	58.634	18.004	16.206	12.727
**MOD**	N	81	81	81	81	81	81	81	81	81	81
	log mean	0.4246	0.8241	1.2022	1.3291	1.0137	0.8347	0.6463	0.3953	0.3655	0.2261
	log SD	0.8807	1.0852	1.3591	1.3358	1.0960	1.0412	0.8419	0.6489	0.5465	0.4048
	log SE	0.0979	0.1206	0.1510	0.1484	0.1218	0.1157	0.0935	0.0721	0.0607	0.0450
	log 95 % CI	0.23 – 0.62	0.59 – 1.06	0.91 – 1.5	1.04 – 1.62	0.77 – 1.25	0.61 – 1.06	0.46 – 0.83	0.25 – 0.54	0.25 – 0.48	0.14 – 0.31
	mean	45.432	88.230	325.926	319.342	89.877	62.551	22.387	8.724	5.500	2.016
	SD	146.572	184.205	587.938	569.225	139.927	107.408	54.646	20.553	15.023	4.304
**Diff**	mean Diff	0.066	0.132	0.277	0.109	-0.001	0.052	-0.056	-0.032	-0.078	-0.069
	log SD	0.798	0.721	0.786	0.625	0.623	0.728	0.699	0.615	0.547	0.422
	90 % CI	0.134	0.164	0.210	0.208	0.172	0.158	0.131	0.102	0.090	0.069
	ratio mean	1.221	1.076	0.829	0.896	0.889	1.159	0.919	0.981	0.796	0.510
Eq. margin 20 %		0.092	0.178	0.268	0.277	0.203	0.172	0.124	0.076	0.065	0.038
Eq. margin 50 %		0.229	0.445	0.670	0.692	0.507	0.430	0.309	0.190	0.163	0.096
Test 20 %		FALSE	TRUE	TRUE	TRUE	TRUE	TRUE	FALSE	FALSE	FALSE	FALSE
Test 50 %		**TRUE**	**TRUE**	**TRUE**	**TRUE**	TRUE	TRUE	TRUE	**TRUE**	**TRUE**	**TRUE**
**Power**											
Eq. margin 20 %		0.00	0.01	0.40	0.75	0.32	0.00	0.00	0.00	0.00	0.00
Eq. margin 50 %		0.14	0.98	1.00	1.00	1.00	0.96	0.75	0.24	0.19	0.00
**Number of nets**											
Eq. margin 20 %		1300.36	281.32	147.68	88.12	162.77	306.82	547.82	1126.46	1202.75	1910.51
Eq. margin 50 %		208.63	45.60	24.23	14.72	26.64	49.67	88.23	180.81	193.01	306.25

B) Pelagic zone of Upper Lake Constance.

		MESH SIZE [mm]									
		**6.25**	**8**	**10**	**12.5**	**15.5**	**19.5**	**24**	**29**	**35**	**43**
**CEN**	N	26	26	26	26	26	26	26	26	26	26
	log mean	0.3331	0.9105	0.8911	0.1784	0.0509	0.0340	0.0000	0.2591	0.0340	0.0445
	log SD	0.5989	0.8782	0.7749	0.4340	0.2593	0.1735	0.0000	0.4401	0.1735	0.2268
	log STD err	0.1175	0.1722	0.1520	0.0851	0.0509	0.0340	0.0000	0.0863	0.0340	0.0445
	log 95 % Ci	0.1 – 0.56	0.57 – 1.25	0.59 – 1.19	0.01 – 0.35	-0.05 – 0.15	-0.03 – 0.1	0 – 0	0.09 – 0.43	-0.03 – 0.1	-0.04 – 0.13
	mean	7.17948718	38.7179487	24.1025641	2.30769231	0.76923077	0.25641026	0	2.3076923	0.25641026	0.51282051
	SD	19.13	69.025	35.331	6.237	3.922	1.307	0	4.191	1.307	2.615
**MOD**	N	26	26	26	26	26	26	26	26	26	26
	log mean	0.1685	0.7817	0.8735	0.0445	0.0680	0.0340	0.1361	0.5062	0.1198	0.0202
	log SD	0.4817	0.8316	0.9323	0.2268	0.2404	0.1735	0.3255	0.4392	0.2554	0.0715
	log SE	0.0945	0.1631	0.1828	0.0445	0.0471	0.0340	0.0638	0.0861	0.0501	0.0140
	log 95 % CI	-0.02 – 0.35	0.46 – 1.1	0.52 – 1.23	-0.04 – 0.13	-0.02 – 0.16	-0.03 – 0.1	0.01 – 0.26	0.34 – 0.68	0.02 – 0.22	-0.01 – 0.05
	mean	3.58974359	23.5897436	40.5128205	0.51282051	0.51282051	0.25641026	1.02564103	3.974359	0.64102564	0.06410256
	SD	11.032	35.036	70.602	2.615	1.812	1.307	2.453	4.424	1.42	0.226
**Diff.**	mean diff	0.165	0.129	0.018	0.134	-0.017	0.000	-0.136	-0.247	-0.086	0.024
	log SD	0.519	0.730	0.494	0.386	0.343	0.240	0.325	0.378	0.295	0.234
	90 % CI	0.152	0.236	0.236	0.097	0.069	0.048	0.066	0.126	0.061	0.046
	ratio mean	0.500	0.609	1.681	0.222	0.667	1.000	-	1.722	2.500	0.125
Eq. margin 20 %		0.050	0.169	0.176	0.022	0.012	0.007	0.014	0.077	0.015	0.006
Eq. margin 50 %		0.125	0.423	0.441	0.056	0.030	0.017	0.034	0.191	0.038	0.016
Test 20 %		FALSE	FALSE	FALSE	FALSE	FALSE	FALSE	FALSE	FALSE	FALSE	FALSE
Test 50 %		**FALSE**	**TRUE**	**TRUE**	**FALSE**	FALSE	FALSE	FALSE	**TRUE**	**FALSE**	**FALSE**
**Power**											
Eq. margin 20 %		0.00	0.00	0.00	0.00	0.00	0.00	0.00	0.00	0.00	0.00
Eq. margin 50 %		0.00	0.32	0.87	0.00	0.00	0.00	0.00	0.14	0.00	0.00
**Number of nets**											
Eq. margin 20 %		1834.84	319.24	135.16	5146.23	14275.17	21375.36	9797.04	418.71	6286.64	22352.88
Eq. margin 50 %		294.14	51.66	22.23	824.07	2284.70	3420.73	1568.20	67.57	1006.54	3577.14

C) Benthic zone of Lower Lake Constance.

		MESH SIZE [mm]									
		**6.25**	**8**	**10**	**12.5**	**15.5**	**19.5**	**24**	**29**	**35**	**43**
**CEN**	N	20	20	20	20	20	20	20	20	20	20
	log mean	0.977215	1.0157	1.394052	1.376893	1.02309457	0.5387582	0.337358	0.144195684	0.3030468	0.1441957
	log SD	0.929921	1.13306	1.325031	1.181476	1.06687855	0.8815723	0.704194	0.44382495	0.6245599	0.443825
	log SE	0.207937	0.25336	0.296286	0.264186	0.2385613	0.1971255	0.157463	0.099242276	0.1396558	0.0992423
	log 95 % CI	0.57 – 1.38	0.52 – 1.51	0.81 – 1.97	0.86 – 1.89	0.56 – 1.49	0.15 – 0.93	0.03 – 0.65	-0.05 – 0.34	0.03 – 0.58	-0.05 – 0.34
	mean	38.667	157.333	248	161.333	272	36	12	2.667	6.667	2.667
	SD	57.236	387.495	561.895	232.671	979.748	98.608	30.455	9.009	13.572	7.797
**MOD**	N	20	20	20	20	20	20	20	20	20	20
	log mean	0.536633	0.74586	1.583356	1.389049	0.95469551	0.6043253	0.337358	0.187732562	0.0884607	0.1996626
	log SD	0.96184	1.18972	1.371664	1.18365	0.92044619	0.8666563	0.704194	0.461613128	0.2722762	0.36909
	log SE	0.215074	0.26603	0.306713	0.264672	0.20581802	0.1937902	0.157463	0.103219833	0.0608828	0.082531
	log 95 % CI	0.12 – 0.96	0.22 – 1.27	0.98 – 2.18	0.87 – 1.91	0.55 – 1.36	0.22 – 0.98	0.03 – 0.65	-0.01 – 0.39	-0.03 – 0.21	0.04 – 0.36
	mean	40	144	397.081	149.333	41.333	25.333	12	2.667	0.667	1.5
	SD	80.93	357.185	101.397	179.491	69.47	55.719	31.759	6.975	2.052	3.148
**Diff.**	mean diff	0.440582	0.26984	-0.1893	-0.01216	0.06839907	-0.0655671	0	-0.04353688	0.2145862	-0.0554669
	log SD	0.750089	0.6766	1.028336	0.906654	0.53918469	0.8471999	0.65537	0.594203096	0.6385703	0.4224934
	90 % CI	0.307051	0.36934	0.426824	0.373327	0.31473334	0.2761622	0.222306	0.143119632	0.1560098	0.1291679
	ratio mean	1.034474	0.91526	1.601133	0.92562	0.15195956	0.7036944	1	1	0.100045	0.5624297
Eq. margin 20 %		0.151385	0.17616	0.297741	0.276594	0.19777901	0.1143083	0.067472	0.033192825	0.0391508	0.0343858
Eq. margin 50 %		0.378462	0.44039	0.744352	0.691485	0.49444752	0.2857709	0.168679	0.082982061	0.0978769	0.0859646
Test 20 %		FALSE	FALSE	FALSE	FALSE	FALSE	FALSE	FALSE	FALSE	FALSE	FALSE
Test 50 %		**TRUE**	**TRUE**	**TRUE**	**TRUE**	TRUE	TRUE	FALSE	**FALSE**	**FALSE**	**FALSE**
**Power**											
Eq. margin 20 %		0.00	0.00	0.00	0.00	0.00	0.00	0.00	0.00	0.00	0.00
Eq. margin 50 %		0.03	0.30	0.45	0.53	0.77	0.00	0.00	0.00	0.00	0.00
**Number of nets**											
Eq. margin 20 %		421.17	253.36	204.99	184.71	127.98	941.52	1616.63	5488.84	4557.23	2586.39
Eq. margin 50 %		67.97	41.12	33.39	30.15	21.08	151.22	259.23	878.89	729.73	414.39

D) Pelagic zone of Lower Lake Constance.

		MESH SIZE [mm]									
		**6.25**	**8**	**10**	**12.5**	**15.5**	**19.5**	**24**	**29**	**35**	**43**
**CEN**	N	9	9	9	9	9	9	9	9	9	9
	log mean	0.6684	0.2268	0.4593	0.0000	0.0000	0.2949	0.0000	0.0983	0.0983	0.0000
	log SD	0.9078	0.4551	0.6961	0.0000	0.0000	0.4423	0.0000	0.2949	0.2949	0.0000
	log SE	0.3026	0.1517	0.2320	0.0000	0.0000	0.1474	0.0000	0.0983	0.0983	0.0000
	log 95 % CI	0.08 – 1.26	-0.07 – 0.52	0 – 0.91	0 – 0	0 – 0	0.01 – 0.58	0 – 0	-0.09 – 0.29	-0.09 – 0.29	0 – 0
	mean	42.962	2.222	8.148	0	-	2.222	-	0.741	0	0
	SD	111.759	4.714	13.24	0	-	3.333	-	2.222	0	0
**MOD**	N	9	9	9	9	9	9	9	9	9	9
	log mean	0.2887	0.2570	0.0000	0.3854	0.0000	0.0000	0.0000	0.0708	0.0865	0.0585
	log SD	0.5773	0.5099	0.0000	0.5782	0.0000	0.0000	0.0000	0.2123	0.2594	0.1161
	log SE	0.1924	0.1700	0.0000	0.1927	0.0000	0.0000	0.0000	0.0708	0.0865	0.0387
	log 95 % CI	-0.09 – 0.67	-0.08 – 0.59	0 – 0	0.01 – 0.76	0 – 0	0 – 0	0 – 0	-0.07 – 0.21	-0.08 – 0.26	-0.02 – 0.13
	mean	4.444	2.962	0	4.444	-	0	-	0.37	0.185	0.185
	SD	9.428	5.879	0	6.667	-	0	-	1.111	0.556	0.367
**Diff.**	mean Diff	0.380	-0.030	0.459	-0.385	0.000	0.295	0.000	0.028	0.012	-0.058
	log SD	0.886	0.455	0.696	0.578	0.000	0.442	0.000	0.341	0.368	0.116
	90 % CI	0.380	0.233	0.265	0.221	0.000	0.169	0.000	0.124	0.134	0.042
	ratio mean	0.103	1.333	0.000	-	-	0.000	-	0.499	-	-
Eq. margin 20 %		0.096	0.048	0.046	0.039	0.000	0.029	0.000	0.017	0.018	0.006
Eq. margin 50 %		0.239	0.121	0.115	0.096	0.000	0.074	0.000	0.042	0.046	0.015
Test 20 %		FALSE	FALSE	FALSE	FALSE	FALSE	FALSE	FALSE	FALSE	FALSE	FALSE
Test 50 %		**FALSE**	**FALSE**	**FALSE**	**FALSE**	FALSE	FALSE	FALSE	**FALSE**	**FALSE**	**FALSE**
**Power**											
Eq. margin 20 %		0.00	0.00	0.00	0.00	-	0.00	-	0.00	0.00	0.00
Eq. margin 50 %		0.00	0.00	0.00	0.00	-	0.00	-	0.00	0.00	0.00
**Number of nets**											
Eq. margin 20 %		1469.24	1516.56	3933.65	3854.41	-	3854.41	-	6974.02	6779.57	6744.03
Eq. margin 50 %		235.65	243.22	629.95	617.27	-	617.27	-	1116.52	1085.41	1079.72
