# Supplementary material for: Obtaining accurate population estimates with reduced workload and lower fish mortality in multi-mesh gillnet sampling of a large pre-alpine lake
Source: PLoS One. 2024 Mar 18;19(3):e0299774. doi: 10.1371/journal.pone.0299774 (PMC10947718; doi:10.1371/journal.pone.0299774)
Supplement: S7 Table — (PDF) [file pone.0299774.s007.pdf]

Table S7. Test of homogeneity of variances in Upper Lake Constance and Lower Lake Constance: PERMDISP comparisons of CEN and MOD nets according to depth stratum and zone.

Depth stratum [m]	Upper Lake Constance			Lower Lake Constance		
	**t**	**P(perm)**	**N**	**t**	**P(perm)**	**N**
Benthic zone						
0–2.9	1.3912	0.233	9	1.2903	0.203	3
3–5.9	1.1347	0.403	11	0.090103	0.946	4
6-11.9	0.61764	0.627	12	0.25539	0.885	4
12-19.9	0.049056	0.97	11	0.48876	0.684	3
20-34.9	0.43222	0.607	10	0.82799	0.698	3
35-49.9	0.78691	0.494	8	3.7581	0.094	3
50-74.9	0.7044	0.621	9			
75-100	0.061437	0.954	11			
Pelagic zone						
6-11.9				1.8489	0.109	3
12-19.9	0.044231	0.973	12	0.47844	0.863	4
20-35	5.1573	0.001	12			
