# Supplementary material for: Obtaining accurate population estimates with reduced workload and lower fish mortality in multi-mesh gillnet sampling of a large pre-alpine lake
Source: PLoS One. 2024 Mar 18;19(3):e0299774. doi: 10.1371/journal.pone.0299774 (PMC10947718; doi:10.1371/journal.pone.0299774)
Supplement: S8 Table — (PDF) [file pone.0299774.s008.pdf]

Table S8. ANOSIM analysis of CEN and MOD nets of each depth stratum. N = number of comparisons (N < 3 shows in best-case trends).

A) Benthic zone of Upper Lake Constance.

Depth stratum [m]	R-statistic	N
0-2.9	-0.07099	9
3-5.9	-0.05169	11
6-11.9	-0.0604	12
12-19.9	-0.01307	11
20-34.9	-0.062	10
35-49.9	-0.08984	8
50-74.9	0.033951	9
75-100	-0.24944	11

B) Pelagic zone of Upper Lake Constance.

Depth stratum [m]	R-statistic	N
6-11.9	0	2
12-19.9	-0.015572	12
20-35	0.029882	12

C) Benthic zone of Lower Lake Constance.

Depth stratum [m]	R-statistic	N
0-2.9	-0.18519	3
3-5.9	0	4
6-11.9	-0.16667	4
12-19.9	0	3
20-34.9	-0.22222	3
35-50	0.074074	3

D) Pelagic zone of Lower Lake Constance.

Depth stratum [m]	R-statistic	N
6-11.9	0.1111	3
12-19.9	-0.19792	4
20-35	-0.25	2
