# Supplementary material for: Obtaining accurate population estimates with reduced workload and lower fish mortality in multi-mesh gillnet sampling of a large pre-alpine lake
Source: PLoS One. 2024 Mar 18;19(3):e0299774. doi: 10.1371/journal.pone.0299774 (PMC10947718; doi:10.1371/journal.pone.0299774)
Supplement: S9 Table — (PDF) [file pone.0299774.s009.pdf]

Table S9. Calculation of labour savings using MOD nets.

A) Total catch, total catch proportion and catch ratio (CEN/MOD) according to mesh size/panel size ratio, and mean catch ratio of CEN and MOD nets in Upper Lake Constance.

		MESH SIZE [mm]									
		**6.25**	**8**	**10**	**12.5**	**15.5**	**19.5**	**24**	**29**	**35**	**43**
**Panel size ratio**			**0.5**				**1**		**2**	**4**	**8**
Benthic zone											
**Total catch**	CEN	108	249	1194	1082	306	164	74	27	21	12
	MOD	69	134	495	485	273	190	68	53	66	49
**Proportion of the total catch**	CEN		81.34				16.81			1.85	
	MOD		62.86				28.22			8.93	
**Catch ratio**		0.64	0.54	0.41	0.45	0.89	1.16	0.92	1.96	3.14	4.08
**Mean catch ratio**			0.51				0.99				
Pelagic zone											
**Total catch**	CEN	28	151	94	9	3	1	0	9	1	2
	MOD	7	52	79	1	2	1	4	31	10	2
**Proportion of the total catch**	CEN		94.63				1.34			4.03	
	MOD		73.53				3.71			22.77	
**Catch ratio**		0.25	0.34	0.84	0.11	0.67	1.00		3.44	10.02	1.00
**Mean catch ratio**			0.39				0.83				

B) Total catch, total catch proportion and catch ratio (CEN/MOD) according to mesh size/panel size ratio, and mean catch ratio of CEN and MOD nets in Lower Lake Constance.

		MESH SIZE [mm]									
		**6.25**	**8**	**10**	**12.5**	**15.5**	**19.5**	**24**	**29**	**35**	**43**
**Panel size ratio**			**0.5**				**1**		**2**	**4**	**8**
Benthic Zone											
**Total catch**	CEN	29	118	185	120	204	27	8	2	5	2
	MOD	15	54	149	56	31	19	9	4	2	9
**Proportion of the total catch**	CEN		64.57				34.14			1.29	
	MOD		78.74				16.95			4.31	
**Catch ratio**		0.52	0.46	0.81	0.47	0.15	0,70	1,13	2,00	0,40	4,50
**Mean catch ratio**			0.56				0.66				
Pelagic Zone											
**Total catch**	CEN	58	3	11	0	0	3	0	1	0	0
	MOD	3	2	0	3	0	0	0	1	1	2
**Proportion of the total catch**	CEN		94.74				3.95			1.32	
	MOD		66.67				0			33.33	
**Catch ratio**		0.05	0.67	0.00	-	-	0	-	1,00	-	-
**Mean catch ratio**			0.24				0				

C) Calculation of labour savings per benthic net using CEN and MOD nets in Upper Lake Constance.

**Panel size ratio**	**0.5**	**1**	**2**	**4**	**8**	**Sum**
Proportion of the total catch in CEN nets	0.8134	0.1681	0.0083	0.0064	0.0037	1
Calculation of MOD nets using catch ratios	0.4148	0.1664	0.0163	0.0203	0.0151	0.63

D) Calculation of labour savings per benthic net using CEN and MOD nets in Lower Lake Constance.

**Panel size ratio**	**0.5**	**1**	**2**	**4**	**8**	**Sum**
Proportion of the total catch in CEN nets	0.6457	0.3414	0.0029	0.0071	0.0029	1
Calculation of MOD nets using catch ratios	0.3616	0.2253	0.0057	0.0029	0.0129	0.6083
