# Supplementary material for: Obtaining accurate population estimates with reduced workload and lower fish mortality in multi-mesh gillnet sampling of a large pre-alpine lake
Source: PLoS One. 2024 Mar 18;19(3):e0299774. doi: 10.1371/journal.pone.0299774 (PMC10947718; doi:10.1371/journal.pone.0299774)
Supplement: S10 Table — (PDF) [file pone.0299774.s010.pdf]

Table S10. ANOSIM analysis of NPUE data (log(x+1) transformed) of benthic nets in each depth stratum of Upper Lake Constance and Lower Lake Constance. Significant differences are marked with an asterisk.

Depth strat. [m]	0-2.9	3-5.9	6-11.9	12-19.9	20-34.9	35-49.9	50-74.9	75-99.9	100-149.9	150-200
Upper Lake Constance										
**3-5.9**	0.16*	-	-	-	-	-	-	-	-	-
**6-11.9**	0.23*	0.05*	-	-	-	-	-	-	-	-
**12-19.9**	0.22*	0.08*	0.05*	-	-	-	-	-	-	-
**20-34.9**	0.39*	0.35*	0.36*	0.15*	-	-	-	-	-	-
**35-49.9**	0.72*	0.73*	0.74*	0.52*	0.10*	-	-	-	-	-
**50-74.9**	0.85*	0.89*	0.93*	0.71*	0.16*	-0.04	-	-	-	-
**75-99.9**	0.86*	0.90*	0.95*	0.75*	0.26*	0.08*	0.03	-	-	-
**100-149.9**	0.82*	0.88*	0.93*	0.68*	0.11*	-0.09	-0.06	-0.06	-	-
**150-199.9**	0.81*	0.86*	0.93*	0.65*	0.04	-0.17	-0.15	-0.15	0.00	-
**200-250**	0.80*	0.86*	0.92*	0.65*	0.02	-0.20	-0.19	-0.17	-0.05	-0.01
Lower Lake Constance										
**3-5.9**	0.14*	-	-	-	-	-	-	-	-	-
**6-11.9**	0.25*	0.02	-	-	-	-	-	-	-	-
**12-19.9**	0.31*	0.21*	0.24*	-	-	-	-	-	-	-
**20-34.9**	0.85*	0.84*	0.86*	0.17*	-	-	-	-	-	-
**35-50**	0.93*	0.97*	0.99*	0.17*	-0.02	-	-	-	-	-
