# Supplementary material for: Obtaining accurate population estimates with reduced workload and lower fish mortality in multi-mesh gillnet sampling of a large pre-alpine lake
Source: PLoS One. 2024 Mar 18;19(3):e0299774. doi: 10.1371/journal.pone.0299774 (PMC10947718; doi:10.1371/journal.pone.0299774)
Supplement: S1 Fig — (PDF) [file pone.0299774.s011.pdf]

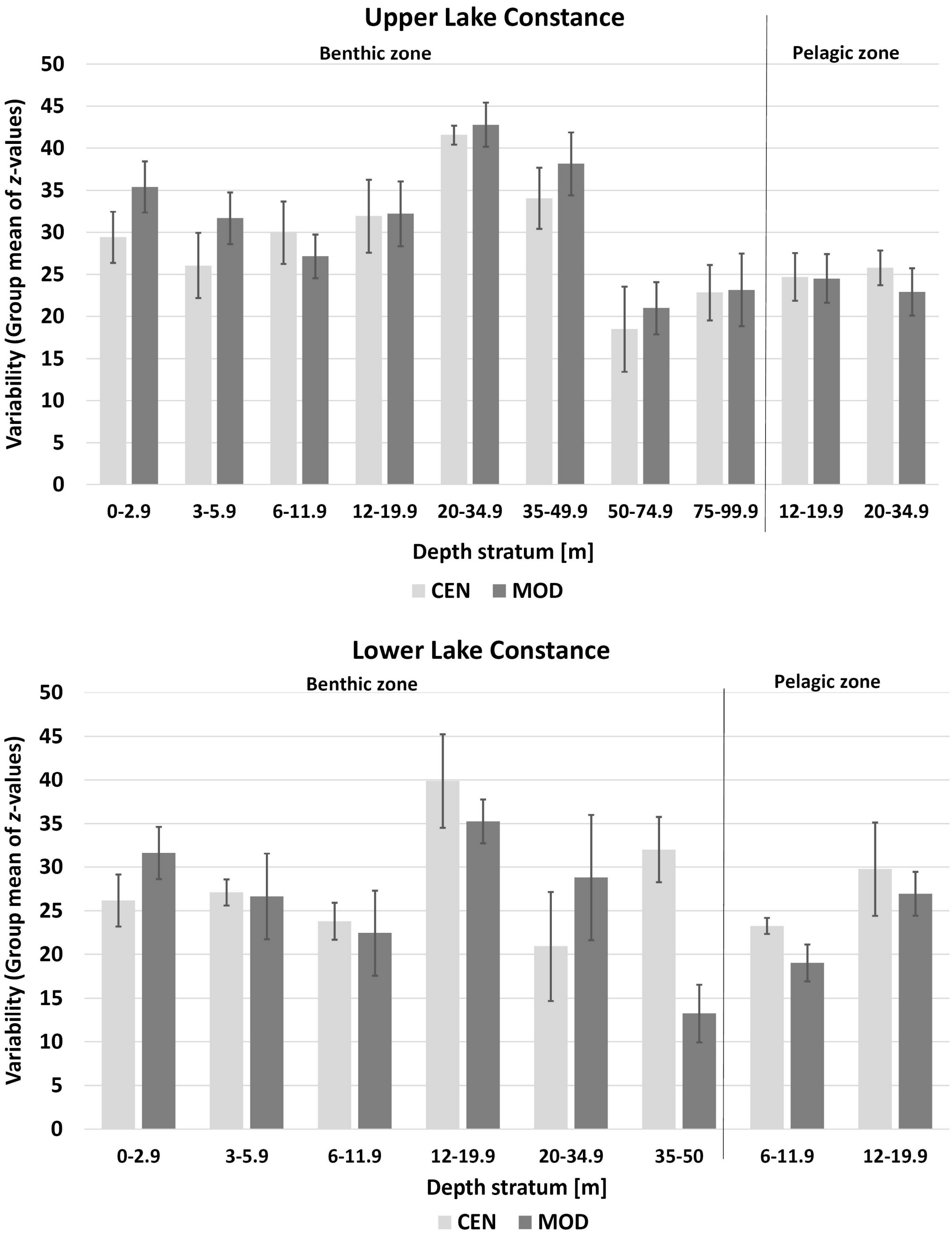

Fig S1. Variation in species composition and number of individuals of depth strata in Upper Lake Constance and Lower Lake Constance expressed as mean *z*-values (PERMDISP) and standard error.
